# Battery-free, wireless soft sensors for continuous multi-site measurements of pressure and temperature from patients at risk for pressure injuries

**DOI:** 10.1038/s41467-021-25324-w

**Published:** 2021-08-24

**Authors:** Yong Suk Oh, Jae-Hwan Kim, Zhaoqian Xie, Seokjoo Cho, Hyeonseok Han, Sung Woo Jeon, Minsu Park, Myeong Namkoong, Raudel Avila, Zhen Song, Sung-Uk Lee, Kabseok Ko, Jungyup Lee, Je-Sang Lee, Weon Gi Min, Byeong-Ju Lee, Myungwoo Choi, Ha Uk Chung, Jongwon Kim, Mengdi Han, Jahyun Koo, Yeon Sik Choi, Sung Soo Kwak, Sung Bong Kim, Jeonghyun Kim, Jungil Choi, Chang-Mo Kang, Jong Uk Kim, Kyeongha Kwon, Sang Min Won, Janice Mihyun Baek, Yujin Lee, So Young Kim, Wei Lu, Abraham Vazquez-Guardado, Hyoyoung Jeong, Hanjun Ryu, Geumbee Lee, Kyuyoung Kim, Seunghwan Kim, Min Seong Kim, Jungrak Choi, Dong Yun Choi, Quansan Yang, Hangbo Zhao, Wubin Bai, Hokyung Jang, Yongjoon Yu, Jaeman Lim, Xu Guo, Bong Hoon Kim, Seokwoo Jeon, Charles Davies, Anthony Banks, Hyung Jin Sung, Yonggang Huang, Inkyu Park, John A. Rogers

**Affiliations:** 1 Center for Bio-Integrated Electronics, Northwestern University, Evanston, IL USA; 2 Department of Mechanical Engineering, Korea Advanced Institute of Science and Technology, Daejeon, Republic of Korea; 3 Department of Electrical and Computer Engineering, University of Illinois at Urbana–Champaign, Urbana, IL USA; 4 Department of Materials Science and Engineering, University of Illinois at Urbana Champaign, Urbana, IL USA; 5 Querrey Simpson Institute for Bioelectronics, Northwestern University, Evanston, IL USA; 6 State Key Laboratory of Structural Analysis for Industrial Equipment, Department of Engineering Mechanics, Dalian University of Technology, Dalian, People’s Republic of China; 7 Ningbo Institute of Dalian University of Technology, Ningbo, People’s Republic of China; 8 Department of Biomedical Engineering, Texas A&M University, College Station, TX USA; 9 Department of Mechanical Engineering, Northwestern University, Evanston, IL USA; 10 Advanced 3D Printing Technology Development Division, Korea Atomic Energy Research Institute, Daejeon, Republic of Korea; 11 Qualcomm Institute, La Jolla, CA USA; 12 Sibel Health Inc, Niles, IL USA; 13 Department of Rehabilitation Medicine, Gimhae Hansol Rehabilitation & Convalescent Hospital, Gimhae, Republic of Korea; 14 Department of Planning and Development, Gimhae Hansol Rehabilitation & Convalescent Hospital, Gimhae, Republic of Korea; 15 Department of Rehabilitation Medicine, Pusan national university hospital, Busan, Republic of Korea; 16 Department of Materials Science and Engineering, KAIST Institute for The Nanocentury (KINC), Korea Advanced Institute of Science and Technology, Daejeon, Republic of Korea; 17 Department of Materials Science and Engineering, Northwestern University, Evanston, IL USA; 18 Department of Mechanical Engineering, Kyung Hee University, Yongin, Republic of Korea; 19 Department of Biomedical Engineering, College of Future Technology, Peking University, Beijing, People’s Republic of China; 20 School of Biomedical Engineering, Korea University, Seoul, Republic of Korea; 21 Interdisciplinary Program in Precision Public Health, Korea University, Seoul, Republic of Korea; 22 Department of Electronic Convergence Engineering, Kwangwoon University, Seoul, Republic of Korea; 23 School of Mechanical Engineering, Kookmin University, Seoul, Republic of Korea; 24 Department of Electrical and Computer Engineering, Northwestern University, Evanston, IL USA; 25 School of Chemical Engineering, Sungkyunkwan University, Suwon, Republic of Korea; 26 School of Electrical Engineering, Korea Advanced Institute of Science and Technology, Daejeon, Republic of Korea; 27 Department of Electrical and Computer Engineering, Sungkyunkwan University, Suwon, Republic of Korea; 28 Biomedical Manufacturing Technology Center, Korea Institute of Industrial Technology (KITECH), Yeongcheon, Republic of Korea; 29 Department of Aerospace and Mechanical Engineering, University of Southern California, Los Angeles, CA USA; 30 Department of Applied Physical Sciences, University of North Carolina at Chapel Hill, Chapel Hill, NC USA; 31 Department of Electrical and Computer Engineering, University of Wisconsin-Madison, Madison, WI USA; 32 NeuroLux, Inc, Glenview, IL USA; 33 Department of Organic Materials and Fiber Engineering, Soongsil University, Seoul, Republic of Korea; 34 Carle Neuroscience Institute, Carle, Physician Group, Urbana, IL USA; 35 Departments of Civil and Environmental Engineering, Northwestern University, Evanston, IL USA; 36 Department of Biomedical Engineering, Northwestern University, Evanston, IL USA; 37 Department of Neurological Surgery, Feinberg School of Medicine, Northwestern University, Chicago, IL USA

**Keywords:** Lifestyle modification, Biomedical engineering, Electrical and electronic engineering, Mechanical engineering, Electronic devices

## Abstract

Capabilities for continuous monitoring of pressures and temperatures at critical skin interfaces can help to guide care strategies that minimize the potential for pressure injuries in hospitalized patients or in individuals confined to the bed. This paper introduces a soft, skin-mountable class of sensor system for this purpose. The design includes a pressure-responsive element based on membrane deflection and a battery-free, wireless mode of operation capable of multi-site measurements at strategic locations across the body. Such devices yield continuous, simultaneous readings of pressure and temperature in a sequential readout scheme from a pair of primary antennas mounted under the bedding and connected to a wireless reader and a multiplexer located at the bedside. Experimental evaluation of the sensor and the complete system includes benchtop measurements and numerical simulations of the key features. Clinical trials involving two hemiplegic patients and a tetraplegic patient demonstrate the feasibility, functionality and long-term stability of this technology in operating hospital settings.

## Abstract (web summary)

Uninterrupted monitoring of pressure and temperature at skin interfaces can help to minimize the potential for pressure injuries in hospitalized or bedridden patients. Here, the authors introduce a soft, skin-mountable sensor that can continuously provide readings via antennas mounted under bedding, and demonstrate the functionality and robustness of the devices on patients.

## Introduction

More than 2.5 million patients in the U.S. develop pressure injuries, cumulatively costing an estimated $9–11 billion for treatment and resulting in 60,000 deaths from their complications each year^1^. Recent pooled estimates over the last decade indicate a prevalence rate of 12.8%, an incidence rate of 5.4 per 10,000 patient per day, and a rate of hospitalized acquired pressure injuries of 8.4%^2^. These injuries correspond to localized damage to the skin and underlying tissue, typically over bony prominences, as a result of prolonged pressures, accelerated by local increases in temperature. The effects on the patients include severe pain, loss of function, reduced mobility and associated risks for complications such as cellulitis^3^, osteomyelitis^4^, and sepsis^5^. The results typically lead to increased economic burdens associated with medical treatments and prolonged hospital stays, along with reduced quality of life^6–9^. The primary mode of prevention involves repositioning (e.g., prone, side-lying or supine) the patient at regular intervals by caregivers or through the use of adjustable mattresses, as outlined by the National Pressure Injury Advisory Panel (NPIAP)^10^. These approaches lead to reducing a risk of developing pressure injuries, but are still not enough to completely prevent their occurrence^11^. The Braden scale scoring system relies on assessments of sensory perception, moisture, activity, mobility, nutrition and friction/shear to define the level of risk^12^. Local changes in skin temperature can serve as an additional indicator^13,14^.

Technologies for continuous measurements of local pressures and temperatures at skin interfaces may provide the basis for an improved, quantitative approach for assessing risk and alerting for the need for preventive action. Previously reported approaches use mattress^15,16^—and textile^17^—integrated pressure sensors. Such technologies do not, however, allow for accurate tracking of ulcer-related variables, including pressure^18^, skin temperature^19^, relative humidity (RH)^20^ and skin blood flow^21,22^ on body locations of interest through the natural course of postural changes. Other emerging schemes include multiple pressure sensors that exploit piezoresistive^23–27^, capacitive^28–31^, piezoelectric^32,33^, and triboelectric^34,35^ effects and mount directly onto the body^24,36^. Reported devices involve, however, some combination of drawbacks in hysteresis, drift and nonlinearity over ranges of pressure of interest. Conventional rigid sensors and wired interfaces are not suitable due to the potential for irritation and damage at the skin interfaces and to limitations in patient mobility, respectively. Furthermore, elevated temperatures that often occur in combination with sustained pressure lead to increased tissue metabolism and oxygen consumption^14^. The associated increases in demand for nutrients and oxygen cannot be fulfilled due to compression of the blood vessels and resulting ischemia. This process increases the rate of skin breakdown^19,37^. Simultaneous monitoring of pressure and temperature, therefore, must be considered.

One of the most promising strategies in this context leverages skin-like wireless device technologies that can noninvasively interface onto relevant regions of the anatomy for continuous monitoring of physiological signals relevant to various patient conditions^38–44^. A specific example involves sensors for pressure and temperature at a variety of skin interfaces, including those to mattresses^40^, prosthetic sockets^42^ or skin-therapeutic compression garments^41^. Customized form factors and operational principles and measurement characteristics can be tailored to the application requirements, but in all cases with comfortable, irritation-free soft contacts to the surface of the skin. The use of battery-free, wireless approaches and protocols enables lightweight devices with the thinnest and most flexible properties. The results support broad possibilities in simultaneous monitoring of pressure and temperature distributions across the entire body at locations of strategic importance, in a manner that is nearly imperceptible to the patient^40,45^.

This paper introduces such a technology, as an advanced version of a previously reported platform^40^ (see Supplementary Note 1 for detail), where the sensor provides greatly enhanced levels of robustness in operation, with responses that are insensitive to bending and shear forces, as required for use in realistic clinical scenarios. Specifically, the pressure sensor can accurately measure pressures across the entire relevant range (<10 kPa) without hysteresis or drift, and with high degrees of linearity, as demonstrated in benchtop studies and verified by numerical simulations of the essential mechanics. Two multiplexed antennas used for power transfer and data communication facilitate accurate, repeatable and continuous monitoring of pressure and temperature from multiple wireless devices mounted on anatomical locations of interest over full-body coverage. Clinical trials involving two hemiplegic patients and a tetraplegic patient demonstrate the feasibility, functionality and long-term stability of the system.

## Results

### Material, device, and system design

**Fig. 1:**
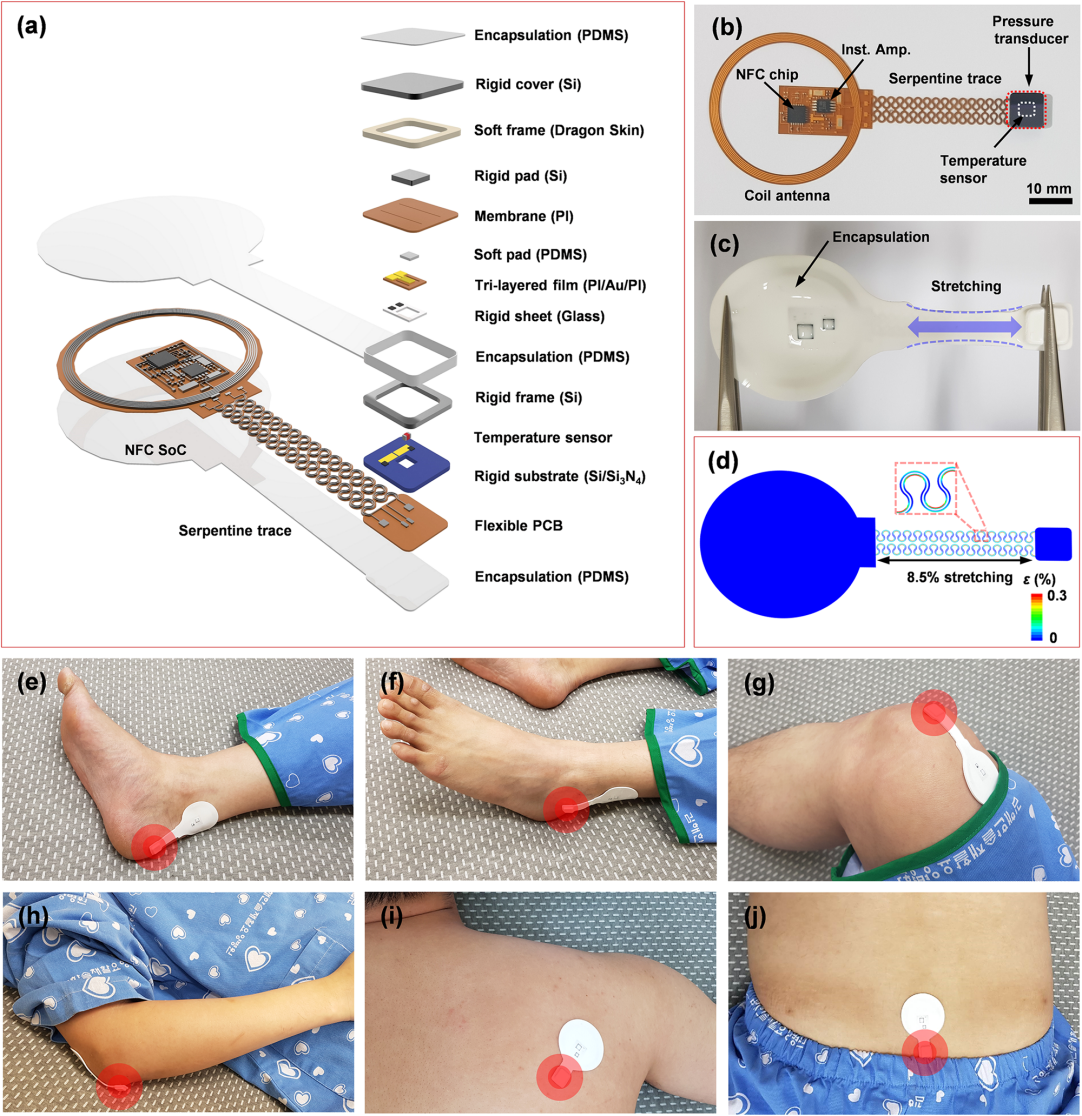
Schematic illustrations and images of a battery-free, wireless pressure, and temperature-sensing platform. **a** Exploded view schematic illustration of the battery-free, wireless pressure and temperature-sensing platform. **b** Photograph of a device before packaging. **c**, **d** Photograph and FEA results for a packaged device in a stretched configuration. **e**–**j** Photographs of devices mounted on body locations that are susceptible to pressure injuries, including the heel, malleolus, knee, elbow, scapulae, and sacrum.

Figure 1a, b presents an exploded view schematic illustration and a photograph of a battery-free, wireless-sensing device that includes a near field communication system-on-a-chip (NFC SoC) and coil antenna, connected via serpentine interconnects to a pressure and a temperature sensor. A flexible printed circuit board (PCB) consisting of patterned layers of copper on a film of polyimide (Cu/PI/Cu, 18/75/18 μm in thickness) serves as a substrate for these components. The receiving antenna, coupled to a primary antenna that is connected to a power module (NFC reader), uses resonant magnetic inductive coupling to harvest power. Furthermore, the combination of the NFC SoC and NFC reader provides wireless communication via standardized ISO 15693 NFC communication protocols. In this way, data from the pressure sensor, amplified using an instrumentation amplifier and digitalized with the analog-to-digital converter (ADC) embedded in the NFC SoC, is wirelessly recorded and logged. Filamentary serpentine electrical traces (length of 6.8–32.2 mm) connect to a distant unit platform that includes a negative temperature coefficient (NTC) thermistor and a resistive pressure sensor designed for reliable operation in the low-pressure regime (~10 kPa). As shown in Supplementary Fig. 1a, a Wheatstone bridge connected to the instrumentation amplifier converts the change in resistance associated with the response of the pressure sensor into a corresponding change in voltage. The amplified analog voltage signal feeds an ADC embedded in the NFC SoC and the results pass into its on-board memory. The NTC forms a voltage divider connected to another ADC of the NFC SoC to record the voltage changes associated with local changes in temperature. The NFC reader then communicates with the devices at 13.56 MHz and pulls the bank of measured data from the memory in each wireless pressure sensor in the field.

Figure 1c shows a photograph of this platform after encapsulation with a thin layer of polydimethylsiloxane (PDMS) that mechanically and electrically protects the electronics and provides a barrier to biofluids. Figure 1c, d illustrates the design to achieve system-level linear elastic (reversible) responses to stretching and other types of deformations, without plastic yielding or fracture in the constituent materials. As shown in Fig. 1d, for stretching of 8.5%, the strains in the Cu layer obtained by the finite-element analysis (FEA) are significantly less than the yield strain (0.3%). This response, together with the elastic (reversible) bending out of the plane (to a bending radius of 7.3 mm), the stretching and twisting (<270˚), ensures soft, irritation-free interfaces to the skin, even in regions of the body that exhibit high levels of curvature such as the edge of the heel, as shown in Supplementary Fig. 2. These materials and schemes in mechanical and electrical design provide the range of robust, low stiffness, elastic responses necessary to accommodate human motions, with little constraint on the underlying skin. Figure 1e–j shows images of these devices mounted on various body locations, including the heel, malleolus, knee, elbow, scapulae and sacrum, that represent areas of high risk for the development of sacral ulcers.

### Design approaches and properties of the pressure sensor

**Fig. 2:**
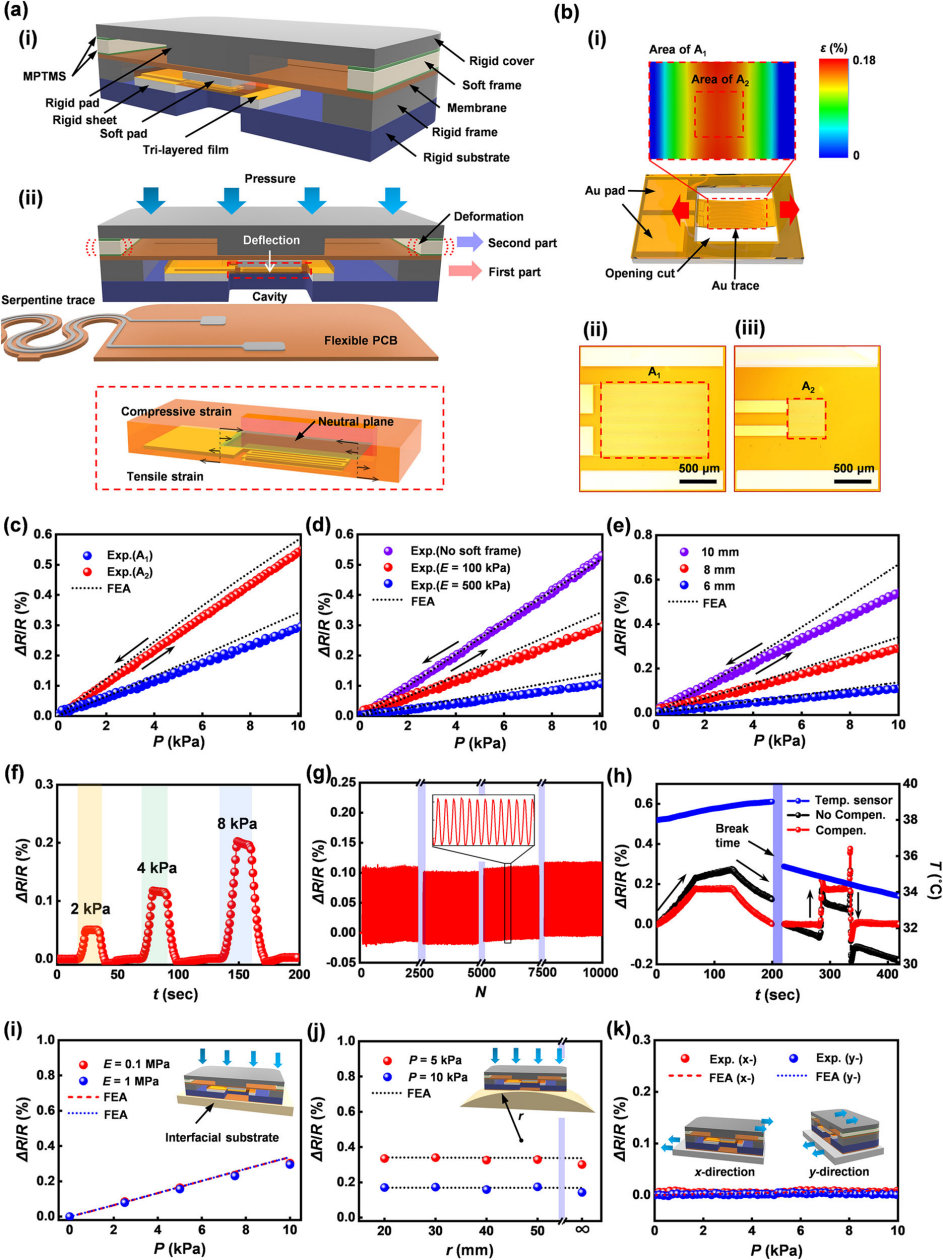
Design features and performance characteristics of pressure sensors based on membrane deflection. **a** (i) Cross-sectional schematic illustration and (ii) operating mechanism of the pressure sensor and its two-part structure. **b** (i) Finite-element analysis results for strain distributions across piezoresistive strain gauges encapsulated with PI films; (ii–iii) photographs of metal traces at different two areas of A_1_ and A_2_ in the tri-layered film. **c** Fractional change in resistance of the piezoresistive strain gauge located in different areas of A_1_ and A_2_. **d** Fractional change in resistance of the pressure transducer with no soft frame and soft frame (*E* = 100 and 500 kPa). **e** Fractional change in resistance of piezoresistive strain gauge at different sizes of device. **f** Response of the pressure sensor against three cyclic loadings of 2, 4, and 8 kPa, respectively. **g** Fractional change in resistance of piezoresistive strain gauge under 10,000 cyclic loading of 4 kPa, respectively. **h** Response of the pressure sensor compensated using measured temperature (NTC) when both pressure and temperature vary. **i** Fractional change in resistance of piezoresistive strain gauge at different values of *E *of the interfacial substrate. **j** Fractional change in resistance of piezoresistive strain gauge at different radii of curvature of interfacial substrate (*E* = 100 kPa). **k** Fractional change in resistance of the piezoresistive strain gauge as a function of the applied shear stresses.

Figure 2a(i–ii) present a cross-sectional schematic illustration of the design and working principle of a pressure sensor that satisfies the demanding requirements for the application considered here, where accurate responses without hysteresis or drift are essential, over a relevant range of pressures without sensitivity to bending, shear or other mechanical deformations. The “Assembly of the pressure sensor” in Methods section provides a design aspect and key material for the assembly procedure for fabricating the pressure sensor, as shown in Supplementary Fig. 3. The pressure sensor has a rigid substrate on the flexible PCB, to prevent bending motions. Electrical traces on the top surface of this substrate connect the Au trace to the serpentine trace. The pressure sensor includes a two-part structure to achieve linear, reversible responses to pressure and to improve robustness under shear loading. A tri-layered film of the first part responds to pressure via deflection-induced tensile strain, resulting in an increase in the resistance. A soft frame of the second part serves to control the sensitivity and operating range via deformation under loadings. A rigid frame and a rigid pad in each part protect the pressure sensor from a damage by excessive loading and a lateral deformation by shear loading, respectively. Supplementary Fig. 4 shows FEA results for the response to shear loading for devices with design A (two-part structure) and design B (one-part structure, without the rigid frame, a membrane of PI and the rigid pad). Design B exhibits large lateral deformations of the soft frame and shear stresses at the interfaces. The comparatively small interfaces stress in design A leads to improved robustness against debonding. The applied pressure allows vertical displacements of the tri-layered film via the deflection, and corresponding tensile strains in the metal trace located below its neutral plane, with an associated increase in its resistance.

The essential properties for the pressure sensor are sensitivity, low hysteresis, high linearity, fast response time, minimal drift, and a measurement range (generally <10 kPa) that matches requirements. Supplementary Fig. 5 shows a photograph of the experimental setup for evaluating the performance of the sensor, as described in detail the Experimental Section. Here, a digital multimeter (USB 4065, NI) and force gauge (M5-05, Mark-10) equipped with a motorized stage (ESM303, Mark-10) yield fractional changes in resistance and corresponding loading values. A calibration process converts these changes in resistance to pressures. Figure 2b(i) shows the distribution of equivalent strains across the tri-layered film (obtained by FEA) for a pressure of 10 kPa. The maximum strain in the Au trace in the tri-layered film is below the yield strain (0.3%). Figure 2b(ii–iii) shows images of two different configurations for the tri-layered film. One exploits traces of Au (linewidths of 7 μm) across an area A_1_ (1 × 1.5 mm^2^) and the other across an area A_2_ (0.5 × 0.5 mm^2^). Strain-induced changes in resistance of the Au trace yield responses with minimal hysteresis and strains that remain below the yield strain of Au across the desired range of pressures. Figure 2c summarizes the sensing performance for a cycle of loading and unloading. The data indicate high linearity (*R*^2^ > 0.99) with negligible hysteresis for these conditions. The magnitude of the fractional changes in resistance can be increased about 1.8 times by decreasing the area of the patterned Au from A_1_ (1.5 × 1 mm^2^) to A_2_ (0.5 × 0.5 mm^2^), as shown in Fig. 2b, consistent with FEA simulation results. On the other hand, two pressure sensors with different initial resistances (10.2 and 20.1 kΩ, respectively) of the metal traces show the same response to pressure, as shown in Supplementary Fig. 6. Figure 2d shows the effects of the soft frame on the sensitivity and operating range. Compression/relaxation of this soft frame structure under loading/unloading controls the response within a lower bound of 0% and an upper bound of 0.6%, as shown in Supplementary Fig. 7 (see Supplementary Note 2). Specifically, the fractional change in resistance without the soft frame is about 0.6% for a pressure of 11 kPa, corresponding to the limits of elastic response of the Au. With the soft frame (elastic modulus (*E*) of 100 or 500 kPa, thickness of 500 μm), this limit occurs at pressures of 20 or 60 kPa, respectively, thereby establishing different ranges of operation, again consistent with simulation results. The effective modulus of the device is related to the properties of the soft frame and the deflection of the membrane, as shown in Supplementary Fig. 8. Miniaturization of the device is another important consideration. Figure 2e displays fractional changes in resistance of the pressure transducer by decreasing the size of device from 10 × 10, 8 × 8, to 6 × 6 mm^2^ with a fixed thickness. Reducing the length of the opening cut (8, 6, and 5 mm, respectively) in the membrane or the ratio of the area of device to the contact area defined by the soft frame reduces the fractional change in resistance, consistent with simulation results.

Figure 2f shows the fractional change in resistance with applied pressure during three cycles of loading and unloading (rates of 0.001 mm/s; sampling rate of 5 Hz). These changes vary as a function of applied pressure with stepwise loadings (2, 4, and 8 kPa, respectively) and return to their initial value with negligible hysteresis (<0.005%) and drift (<0.01%) after unloading. Supplementary Fig. 9 shows the average response of 10 pressure sensors to applied loadings of 3 kPa and 6 kPa, respectively. These results mean that the sensors have reasonable reproducibility, as described in Supplementary Note 3. The results in Fig. 2g indicate long-term stability and mechanical durability under 10,000 repeated cycles to pressures of 4 kPa. The drift in signal is small, typically dominated by slight changes in temperature. As indicated in Supplementary Fig. 10, the resistance changes not only with pressure, but also with temperature due to the temperature dependence of the resistivity of the Au. This effect can be removed using measurements of temperature from the NTC component located at the center of the pressure sensor and the following calibration equation, (Δ*R*/*R*)_c_ = (Δ*R*/*R*)_m_ − *a*Δ*T*, where (Δ*R*/*R*)_c_ and (Δ*R*/*R*)_m_ are the compensated and measured fractional change in resistance of the pressure sensor, respectively, and *a* is the calibration factor. Figure 2h shows responses (black color) to applied loads during changes in temperature (blue color) at the same time. The pressure sensor presents the response (left) to gradual loading/unloading, with a constant response of 6 kPa when the temperature increases from 38 to 39 °C. The device then shows a response (right) under instantaneous loading/unloading, at a load of 6 kPa as the temperature decreases from 35.4 to 33.7 °C. Responses (red color) compensated using the measured temperature effectively isolate the effect of pressure. These results demonstrate that the temperature effect on the response in the pressure sensor can be eliminated using data obtained from temperature sensor (NTC). Beyond its importance in compensation, the temperature itself is a separately interesting parameter in the context of monitoring for development of sacral ulcers^14^.

The pressure-sensing mechanism must also be insensitive to the elastic moduli of the skin and underlying tissues, as well as those of the contacting surfaces. In Fig. 2i, j, the use of flat and curved elastomeric substrates with different moduli provides the basis for evaluating the sensing performance. Figure 2i illustrates that responses on substrates of a low modulus silicone (Dragon Skin; *E* = 100 kPa) and PDMS (*E* = 1 MPa), as shown in Supplementary Fig. 11, are similar. Figure 2j compares the responses measured on different radii of curvature (*r*) of the low modulus silicone substrate under loading of 5 and 10 kPa, as shown in Supplementary Fig. 12. The responses represent the average fractional changes in resistance: 0.16% with standard deviation of 0.01% and 0.32% with standard deviation of 0.02% for 5 and 10 kPa, respectively. Responses to shear forces can also be important. The devices with designs reported here are insensitive to shear along orthogonal directions, as summarized in the results of Fig. 2k, consistent with simulation. In addition, the pressure sensor should be insensitive to RH to enable continuous, reliable measurements in realistic clinical conditions. Supplementary Fig. 13 shows responses of the pressure sensor at different values of RH across the range from 20 to 80% at a temperature of 30 °C. The results demonstrate that the pressure sensor is insensitive to RH under these conditions. Also, Supplementary Fig. 14 exhibits invariant responses at a fixed RH of 80% for 12 h.

The clinical standard for measuring pressures against soft tissues relies on an inflatable bladder and a pneumatic gauge (Picopress, Microlab Elettronica SAS). The data in Supplementary Fig. 15 indicate that the pressure sensor returns readings that compare favorably with this standard for 40 locations across the body of a subject lying on a mattress. In addition, the deformable serpentine traces facilitate attachment to locations of interest with negligible fractional change (Δ*R*/*R* < 0.004%) in resistance of the pressure sensor during 1000 repeated cycles of stretching (8%), bending (7 mm) and twisting (180°), in Supplementary Fig. 16. These results illustrate the stability of operation under mechanical deformations after integrating the pressure sensor into the wireless platform.

### Multiplexed antenna system for full-body-scale power delivery and data communication

**Fig. 3:**
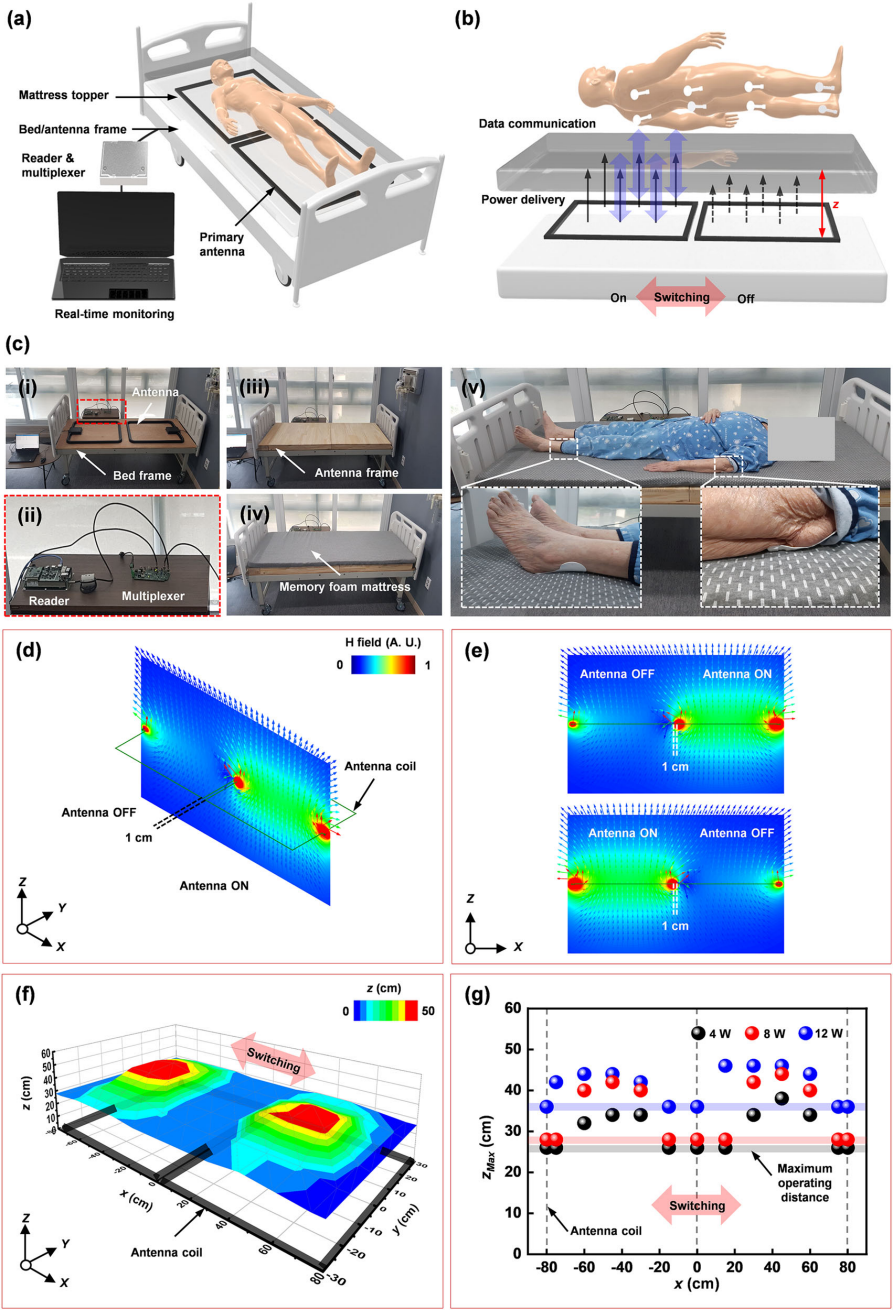
Schematic illustrations and photographs of a battery-free, wireless electronic-sensing system for pressure and temperature measure at the skin interfaces while lying on bed, and associated characteristics of the wireless interface. **a** Schematic illustration of the overall system, which includes two primary antennas, a multiplexer, an antenna reader and a laptop computer for real-time monitoring. **b** Schematic illustration of the antenna embedded between the bed frame and the antenna frame below a mattress topper for delivering power to and reading data from multiple, battery-free devices. **c** Photographs of multiplexed primary antennas integrated with a hospital bed, including an antenna frame and memory foam mattress. **d** Magnetic field distribution for the multiplexed antenna configuration. **e** Computed magnetic field strength and direction as a function of vertical distance away from the XY plane at different RF powers of 4, 8, and 12 W, respectively. **f** Measurements of operating range for the two multiplexed antennas. **g** Comparison of maximum operating range over the two multiplexed antennas at different RF powers (4, 8, and 12 W).

Figure 3a, b illustrates a complete system for continuous and simultaneous monitoring of pressure and temperature across many locations at the interface between the skin and a mattress for a subject lying on a hospital bed. Sensors mounted at various positions yield real-time readings of pressure and temperature in a fast (e.g., data from eight sensors each second) sequential readout scheme based on NFC protocols from a pair of primary antennas interfaced to a multiplexer and an NFC reader located at the bedside. Figure 3c(i–iv) shows pictures of this hardware installed in a hospital room, with primary antennas (62 × 83.8 × 2.6 cm) supported by the frame of the bed, and underneath the topper to provide full-body coverage for a typical subject (75-year-old male, 55 kg, 150 cm; Fig. 3c(v)). Figure 3d, e shows the results of computations of the magnetic field distribution, strength and direction of the multiplexed primary antennas in the XZ plane (where Z is out of the plane of the mattress), which shows small electromagnetic interference during antenna operation. This multiplexed antenna system supports a magnetic field distribution for power transfer and data communication that can cover the area of the clinical bed in a sequential mode. Figure 3f shows the measured maximum operating range (*z*_max_) of a device away from the XY plane for this type of multiplexed antenna setup with no gap to provide a coverage comparable to the scale of the human body (~170.4 cm). Figure 3g shows the results of experimental measurements of operating range for the wireless device under the multiplexed antennas at different radio frequency (RF) powers. The multiplexed antenna can operate over ranges of 26, 28, and 36 cm in the Z direction to cover the entire area in the XY plane depending on the antenna power of 4, 8, and 12 W. The computed magnetic field distribution along the central axis of the antenna shown in Supplementary Fig. 17 supports these experimental results as a function of distance out of the plane of antenna coil for different RF powers. The computations in previous work^40,43^ also indicate that operation falls within guidelines outlined by Federal Communications Commission (FCC) (47 CFR Part 1.1310 and 15) and Food and Drug Administration (FDA) in terms of both the specific absorbed radiation and the maximum permissible exposure. The specific values are lower than limits for the various cases considered.

Supplementary Fig. 18 summarizes the overall operation as a block diagram that includes three subsystems: (1) the wireless, battery-free device to convert analog signals of pressure and temperature variations into digital signals for data communication; (2) the multiplexed primary antenna and the antenna reader for wirelessly delivering power to the device and receiving digital signals of pressure and temperature from the NFC chip, respectively; (3) the real-time visualization of digital signals using a software interface on a PC followed by the post processing for data acquisition and classification.

### Performance of the battery-free, wireless-sensing platform

**Fig. 4:**
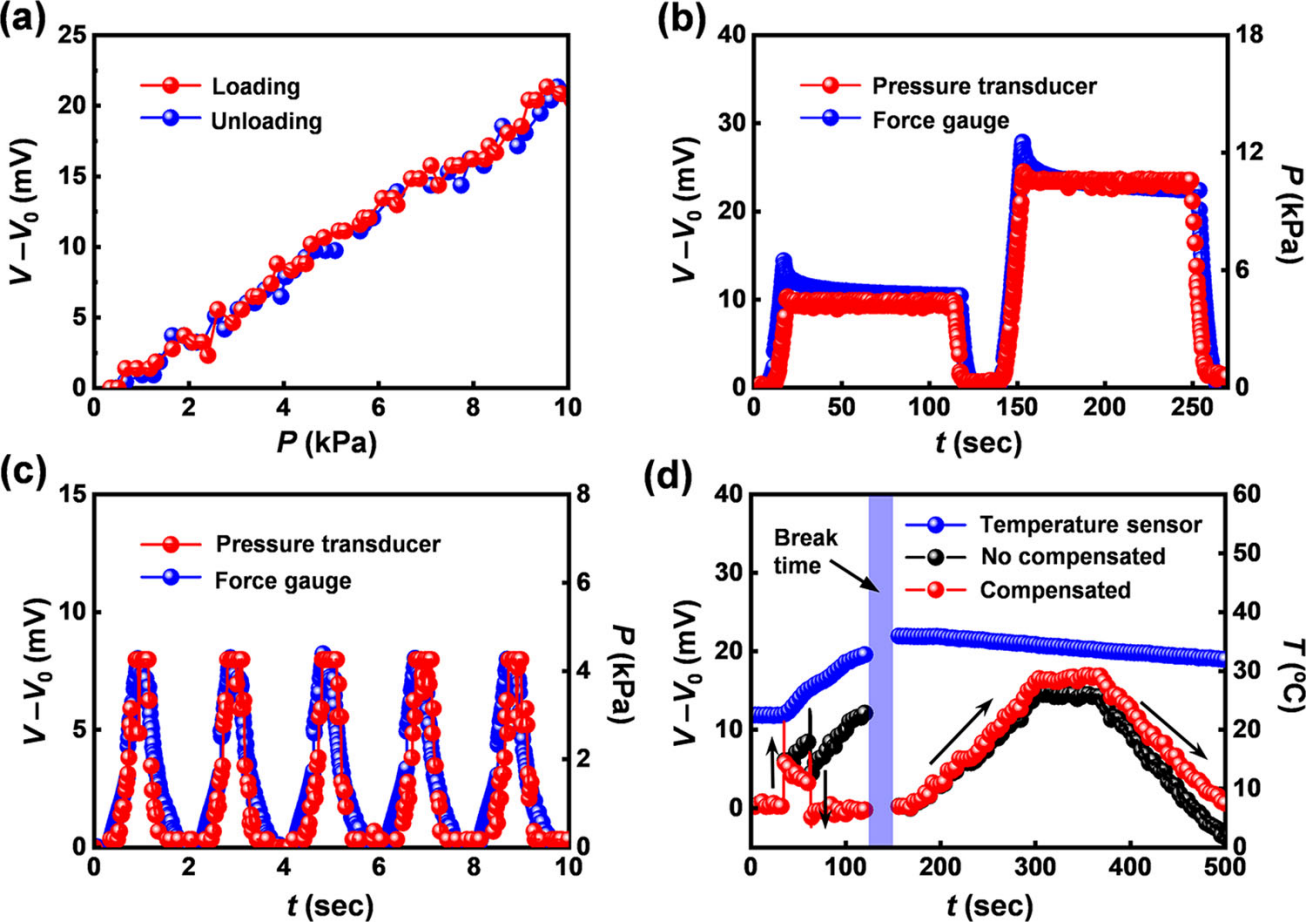
Characteristics of the wireless, battery-free pressure and temperature sensor. **a** Response of the wireless pressure sensor under pressure loading and unloading. **b** Change of the ADC value from the NFC SoC under constant loadings of 4.8 and 11.8 kPa, respectively. **c** Change of the ADC value for five cycles of loading/unloading. **d** Change of ADC values of the wireless pressure sensor compensated using measured temperature (NTC) when both pressure and temperature vary.

The sensor designs and the RF readout schemes described in previous sections serve as the basis for a complete system that allows multi-point pressure and temperature-sensing in a wireless, battery-free mode. Supplementary Fig. 19 shows the quality (Q) factor of a wireless antenna for stable operation of the sensing platform. Figure 4a presents the change of the ADC values of the NFC SoC in response to gradually applied pressures, acquired through the complete wireless system. The data show a linear response (R^2^ > 0.99) with minimal hysteresis (<0.1%) upon loading and unloading, which enables the change of ADC values collected from the wireless pressure sensor to yield pressure, as described in Supplementary Note 4.

Figure 4b, c shows time domain responses of the change of the ADC value under constant (4.8 and 11.8 kPa, respectively) and cyclic loading (0–4.3 kPa). The drift of the ADC values is <0.2% during compression/release cycles. In Fig. 4d, the change of the ADC values from the pressure sensor can be compensated with temperatures measured using the temperature sensor (NTC), as described in Supplementary Note 4. The temperature effect on the ADC values of pressure sensor can therefore be removed using measurements from the NTC component, using the following calibration equation, (ΔV)_c_ = (ΔV)_m_ − *β*ΔT_NTC_, where (ΔV)_c_ and (ΔV)_m_ are the compensated and measured changes in voltage of pressure sensor, and (ΔT)_NTC_ is the temperature change measured using the NTC component, respectively, and *β* is the calibration factor. These findings suggest that the ADC value obtained using the above equation and measured temperature values with *β* = 3 eliminates the temperature effect on the response of the pressure sensor in the context of the applications envisioned here. Results in Fig. 4d show responses (black color) to applied loads during changes in temperature (blue color) at the same time. The pressure sensor presents the response (left) to instantaneous loading/unloading of 3 kPa as the temperature increases from 22.4 to 33.0 °C. The device then shows a response (right) under gradual loading/unloading, with a constant load of 9 kPa as temperature increases from 35.9 °C to 31.9 °C. Responses (red color) compensated using measured values of temperature isolate changes in pressure. These results confirm that the temperature effect on the response of the pressure sensor can be eliminated using data obtained from temperature sensor (NTC). Supplementary Fig. 20 shows the change of the ADC values of the NFC SoC in response to 10,000 repeated cycles of stretching (8%), bending (radius of 7 mm) and twisting (180˚). The large deformations of the serpentine traces lead to small or negligible changes of the ADC values, since the serpentine traces have initial resistances (*R*_Ser_ = 1.4 Ω) and maximum changes in resistance (Δ*R*_Ser_ = 0.4 Ω) much smaller than those of *R*_Pre_ = 20 kΩ and *R*_Tem_ = 100 kΩ, as shown in Supplementary Fig. 1. Supplementary Fig. 21 shows the change of the ADC values of the NFC SoC in response to operating in biofluid (e.g., water or sweat) for 1 h. Since the encapsulation layer of PDMS serves as an effective water barrier under these conditions, the device exhibits stable operation under these conditions.

### Real-time monitoring of pressure changes associated with different lying postures

**Fig. 5:**
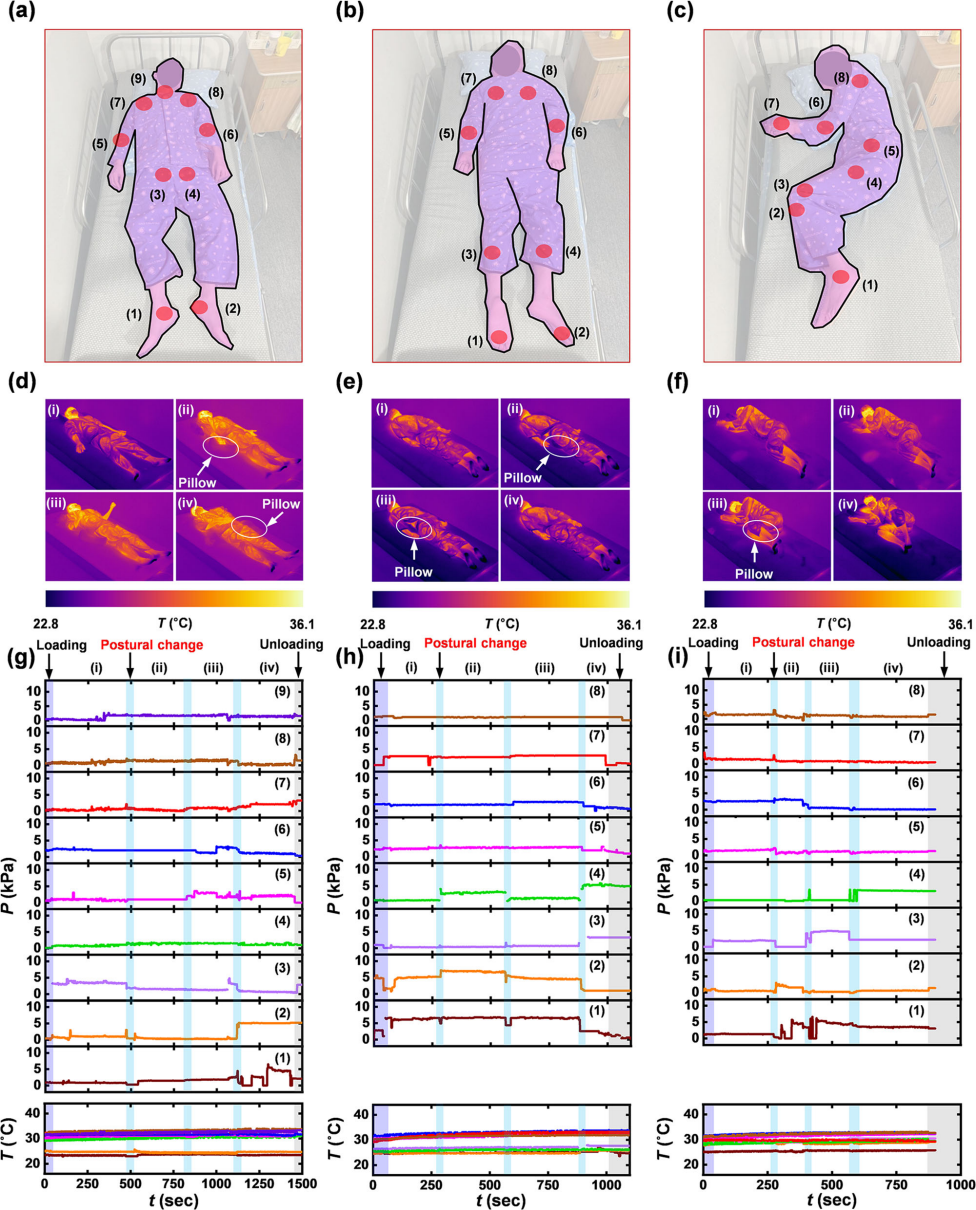
Continuous measurements of pressure and temperature from a healthy subject (30-year-old male, 72 kg, 180 cm) using the wireless-sensing platform at different lying postures, including supine, prone and side-lying positions, respectively. **a**–**c** Photograph and schematic illustration of the subject lying on bed. The red discs highlight the locations of the sensors. **d**–**f** IR images of changes in posture of the subject lying on bed with a pillow at different positions. **g**–**i** Results from continuous measurements of pressure and temperature from each of the sensors.

Validation trials involve continuous measurements of pressure and temperature at multiple locations across the body of a healthy subject (male, 30-year-old; mass, 72 kg; skin temperature, ~36 °C) lying on a bed in a hospital room. Figure 5a–c shows device placements for supine, prone and side-lying positions, selected according to those regions known to be susceptible for formation of pressure injuries due to protruding aspects or bones. For these lying postures, pressure is a common type of force formed by body weight compared to shear force, as described in Supplementary Note 5. The locations include heels (1–2), sacrum (3–4), elbows (5–6), scapulae (7–8) and neck (9) in the supine position, and toes (1–2), knees (3–4), elbows (5–6) and acromion process (7–8) in the prone position, and lateral malleolus (1), lateral knee (2–3), greater trochanter (4), iliac crest (5), elbow (6), wrist (7), acromion process (8) in the side-lying position. The form factor of the devices enables conformal contact with the skin, without discomfort for all of these postures and mounting locations. Figure 5d–f shows infrared camera (IR) photographs of changes in posture at different times through the course of the study. Figure 5g–i presents the results of continuous recordings (sampling rate of 0.5–1 Hz) of temperatures and pressures at each of the different mounting locations. The devices facilitate not only continuous measurement of fluctuating pressure associated with movements, but also decreasing (or increasing) local pressures at selected locations, with addition of pillows by a research staff at the skin-mattress interfaces. The results yield pressures and duration times at mounting locations of interest, including effects of changes in posture at different lying positions. The maximum values of pressure are 6.5 kPa at the right heel in the supine position, 7.2 kPa at the left toe in the prone position and 6.6 kPa at the greater trochanter in the side-lying position. Also, the data from the temperature sensors capture changes in skin temperature with movement for each lying posture. The temperatures are in the range of 22.9–34.0 °C in the supine position, 19.7–34.0 °C in the prone position, 24.9–33.1 °C in the side-lying position. The average skin temperatures measured from the heels, toes and lateral malleolus for each lying posture are 24.6 °C, 24.8 °C, and 25.5 °C, respectively. As expected, these regions, far away from the heart and the core body, have lower temperatures than those obtained at other mounting locations.

In Fig. 5g–i, discontinuous periods of data collection during changes in posture of the subject depend on large tilt angles of the receiver coil and movement of the receiver antenna out of the region encompassed by primary antenna. These issues can be minimized by shaped designs of the receiver antenna and sufficiently large antennas for covering the entire size of clinical bed, as described in Supplementary Note 6.

Supplementary Fig. 22a shows photographs of a subject with 4 devices mounted near the sacrum in the prone position. Red disks mark the mounting locations of 4 sensors placed in proximity to one another with spacings of (ii) 24 mm and (iii) 16 mm. Supplementary Fig. 22b presents data obtained from continuous measurements of pressure and temperature from each of the sensors while the subject is in a supine position. For spacings of 24 mm and 16 mm, maximum values of pressure are 4.4 and 4.9 kPa at the mounting location of (1), respectively. Also, the temperatures are in the range of 22.7–28.0° for spacings of 24 mm and in the range of 26.6–29.6° for spacings of 16 mm. This approach captures the pressure distribution and its change for locations where it is difficult to define the exact area subjected to body weight pressure in Supplementary Note 7.

### Continuous monitoring of pressure changes for extended periods of time

**Fig. 6:**
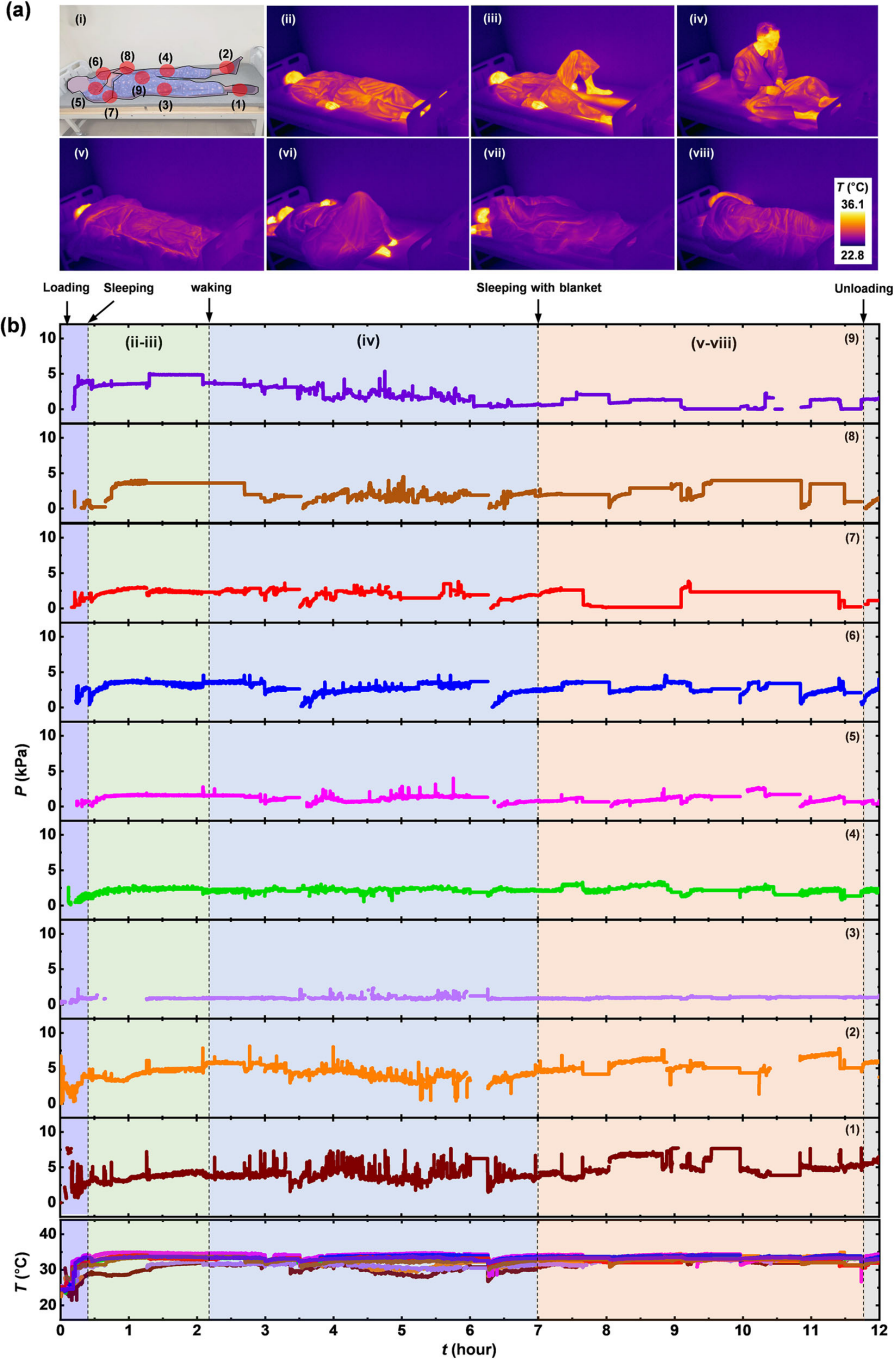
Continuous measurements of pressure and temperature from a healthy subject (30-year-old male, 72 kg, 180 cm) using the wireless-sensing platform during an extended period of time. **a** Photograph of the subject with red discs to mark the mounting locations of the sensors and IR images of changes in posture of the subject lying on bed during biphasic sleep. **b** Results from continuous measurements of pressure and temperature from each of the sensors.

Extended studies rely on continuous measurements of pressure and temperature at multiple locations across the body of healthy subjects for up to 12 h. Figure 6a(i) illustrates 9 mounting locations, including the heel (1–2), the thigh (3–4), the scapulae (5–6), the elbow (7–8), and the sacrum (9). The subject in this case shows a biphasic sleep pattern that involves sleeping for 2 h, waking for a couple of hours and then returning to sleep for several hours. The IR photographs in Fig. 6a(ii–viii) show changes in posture at different times during sleep. Figure 6b shows representative results for temperature and pressure. During the first sleeping period, pressure remains relatively constant with some variations that follow from fine movements. During the waking period, the pressures fluctuate significantly due to changes in posture from lying on the side, to sitting, standing, moving, walking and stretching. During the second sleep period (under a blanket), the pressure changes in a smooth, continuous manner depending on spontaneous changes in posture. The maximum value of pressure is 8.1 kPa at the right heel in the supine posture. The skin temperatures at mounting locations range from 21.5 to 34.9 °C. Cumulative data over a period of time support proper repositioning strategies by considering sleep patterns for individual subjects. In addition, this approach offers capabilities not only for preventing pressure injuries for patients with chronic paralysis, but also for patients who have an acute reduction of activity at specific areas due to accidents, surgery or other medical devices.

### Feasibility trials with hemiplegic and tetraplegic subjects

**Fig. 7:**
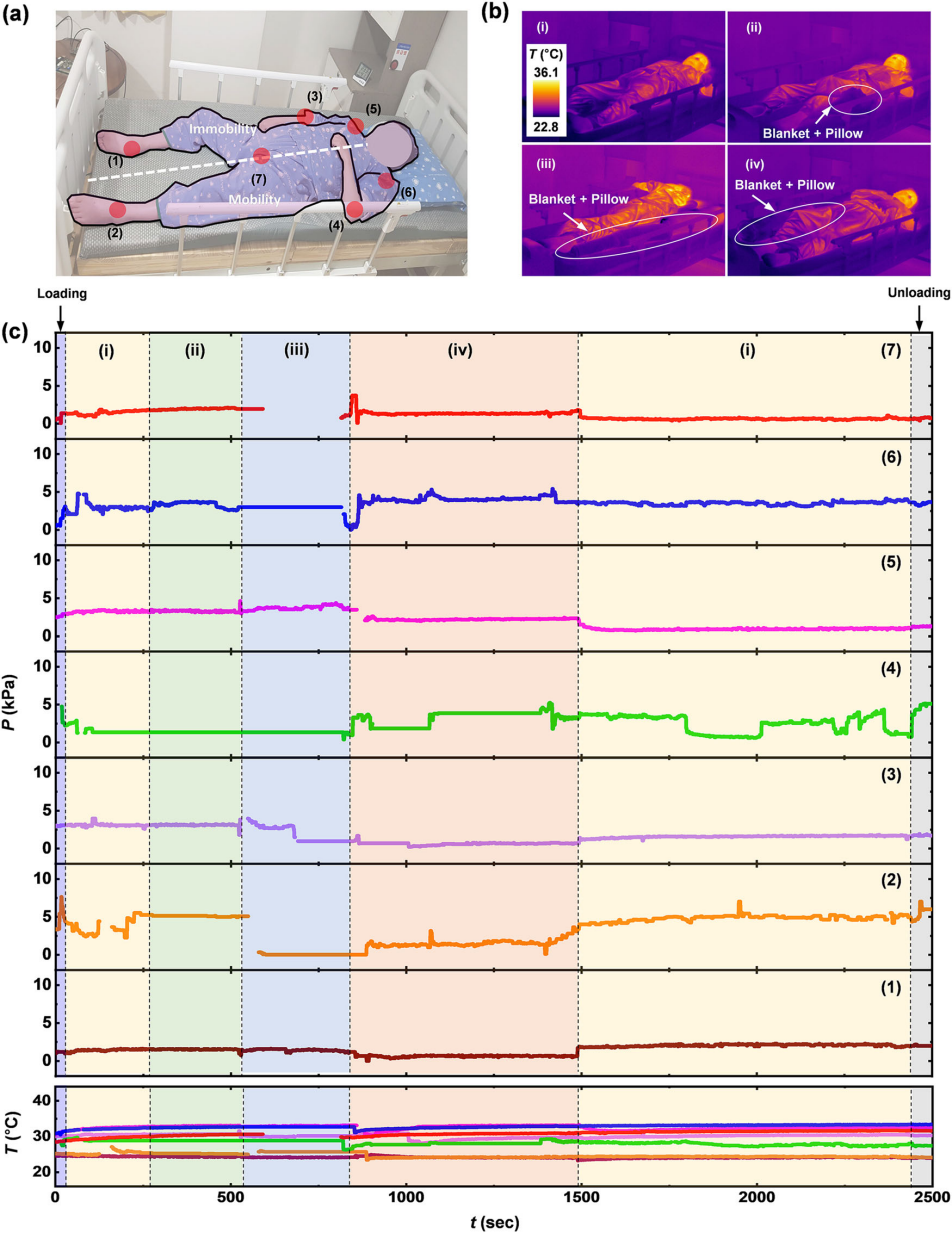
Continuous measurements of pressure and temperature from a subject with right hemiplegia (47-year-old female, 62 kg, 160 cm) using the wireless-sensing platform. **a** Photograph of the subject with red discs to mark the mounting locations of the sensors. **b** IR images of changes in posture of the subject lying on bed with a pillow at different positions. **c** Results from continuous measurements of pressure and temperature from each of the sensors.

Continuous measurements on two hemiplegic patients and a tetraplegic patient illustrate applications of this system with subjects prone to develop pressure injuries. The hemiplegic patient is a 47-year-old, female, with a height of 160 cm, a weight of 62 kg, a total score of 12 points on the Braden scale, blood pressure of 110/70 mmHg, albumin (3.6 g/dl), hemoglobin A1c (5.7%), considered to be at risk. Figure 7a illustrates the seven mounting locations for this case, including heels (1–2), elbows (3–4), scapulae (5–6), and sacrum (7). Figure 7b(i–iv) shows IR photographs of changes in posture selected by the clinical staff for local pressure relief, with addition of blankets and pillows at the skin-mattress interfaces within 2 h for prevention of pressure injuries according to guidelines from NPIAP. Figure 7c summarizes continuous recordings of pressure and temperature at 7 mounting locations against postural changes in the supine position. The data show fine movements of the patient in plots of (1), (3), (5) and (7) due to right-side paralysis, and spontaneous changes in posture caused by the patient in plots of (2), (4) and (6) with activity on the left side, in addition to a change in posture induced by the clinical staff. The patient has the ability to hold the bedside rails with the left hand during repositioning with help of clinical staff, as shown in Supplementary Fig. 23. However, the patient’s own activity is not sufficient to relieve the pressure on the right side of body by spontaneous changes in posture. As expected, the hemiplegic patient has a high risk for developing pressure injuries on locations with low levels of activity, without repositioning. In this respect, the system could guide repositioning strategies. The maximum values of pressure (7.6 kPa) appear at the sacrum. The temperatures on the mounting locations are in the range of 23.2–34.5 °C. Local increases in temperature can accelerate skin necrosis under ischemic anemia, as each increase of 1 °C in skin temperature leads to an increase of ~10% in tissue metabolic requirements^46,47^.

**Fig. 8:**
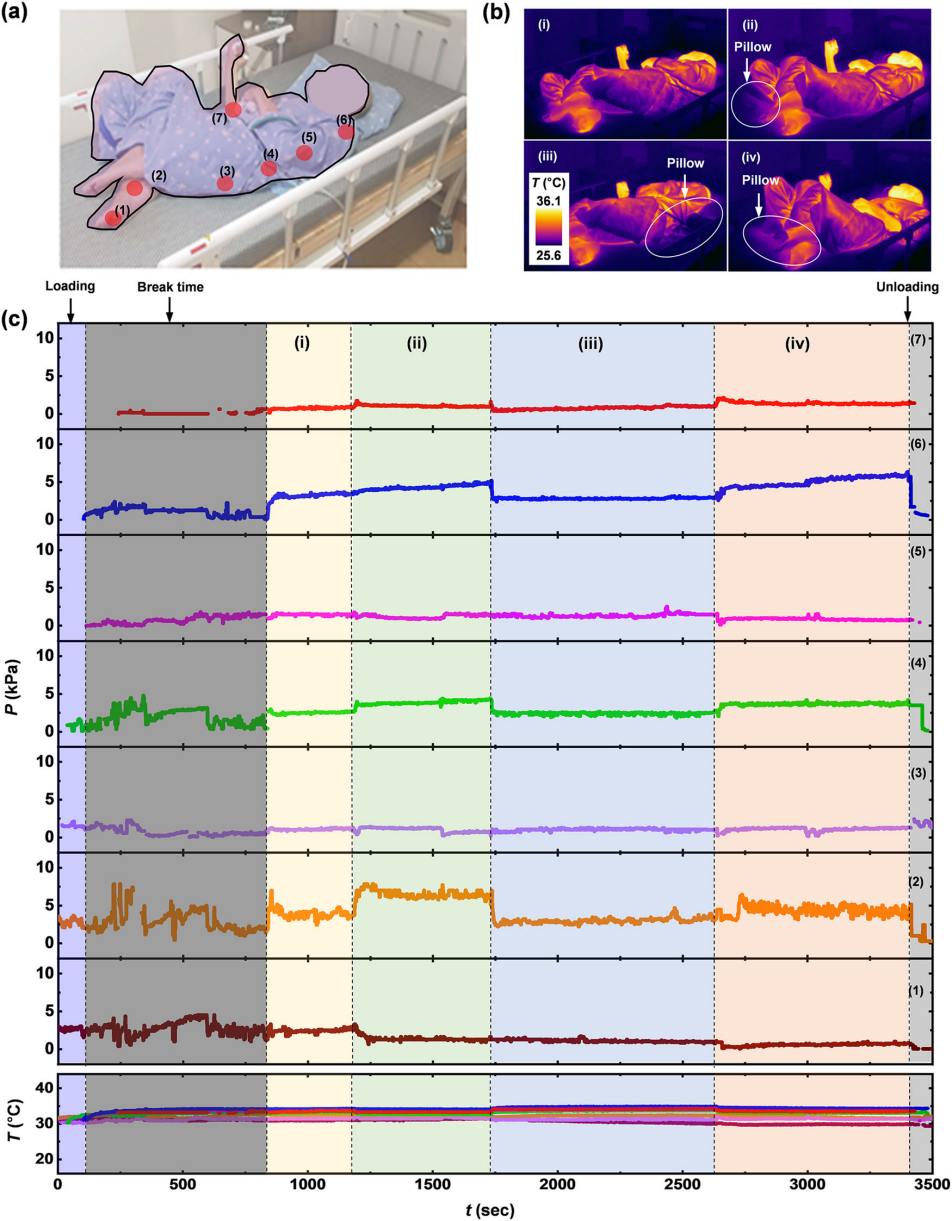
Continuous measurements of pressure and temperature from a subject with general paralysis (83-year-old male, 40 kg, 150 cm) using the wireless-sensing platform. **a** Photograph of the subject with red discs to mark the mounting locations of the sensors. **b** IR images of changes in posture of the subject lying on bed with a pillow at different positions. **c** Results from continuous measurements of pressure and temperature from each of the sensors.

The tetraplegic patient is an 83-year-old, male, with a height of 150 cm, a weight of 40 kg, a total score of 12 points on the Braden scale, blood pressure of 100/60 mmHg, albumin (3.7 g/dl), hemoglobin A1c (5.8%), also in a high risk category, and with spasticity. Figure 8a illustrates 7 mounting locations including the toe (1), the malleolus (2), the greater trochanter (3), the iliac crest (4), the ribs (5), the acromion process (6), the elbow (7). Figure 8b(i–iv) shows IR photographs of changes in posture, induced by clinical staff to reduce local pressure, as with the previous case. Figure 8c highlights results of continuous recordings of temperatures and pressures at seven mounting locations in the side-lying position. The data display fine movements of the patient at almost all mounting locations, except in the plot of (2), if there is no change in posture induced by the clinical staff. The results suggest a high risk for the tetraplegic patient to develop one or more pressure injuries. Also, the spasticity that often exists in these patients complicates clinical management. For this reason, effective repositioning based on quantitative data is important. In this respect, the system could establish a quantitative basis for the effective repositioning at all of mounting locations from pressure and duration time as well as the level of physical activity. The malleolus shows the maximum pressures (7.8 kPa). The mounting locations of the subject show the temperatures are in the range of 27.4–34.9 °C. As prolonged and untreated spasticity in the lying posture leads to restriction of blood circulation and, often, skin damage by friction, measurements of associated changes in temperature are important to capture.

The third trial involves a hemiplegic patient due to basal ganglia hemorrhage, a 61-year-old, male, with a height of 170 cm, a weight of 57 kg, a total score of 15 points on the Braden scale, blood pressure of 120/80 mmHg, albumin (4.1 g/dl), hemoglobin A1c (5.3%). Supplementary Fig. 24a(i) illustrates 7 mounting locations, including the heel (1–2), the elbow (3–4), the scapulae (5–6) and the sacrum (7). The IR photographs in Supplementary Fig. 24a(ii–vi) show changes in posture during a sleep period (11pm-5am) with a blanket covering the body, as shown in Supplementary Fig. 24a(ii–iii). Before sleep, the patient exhibits significant changes in posture, from partially side-lying, to sitting and standing. Supplementary Fig. 24b indicates continuous recordings of temperatures and pressures in the supine position. Before sleeping, sensors at all locations indicate significant fluctuations in pressure, consistent with expectation. The pressure changes in a smooth, continuous manner during sleep, including variations that follow from fine movements. The low level of activity while sleeping, compared to that collected while awake, could lead to increased risks for pressure injuries. These data form the basis for assessing risks for pressure injuries and for alerting staff of the need for proper preventive action. Maximum value of pressure is 6.6 kPa at the heel in the supine posture. The pressures are lower for this patient than the others due to changes in body position that shift pressure away from the sensors. The temperatures are in the range of 26.4–35.7 °C. Supplementary Fig. 25 shows photographs of the right elbow and right heel after removing the devices. The skin shows no redness or other signs of irritation after continuous monitoring for 6 h.

## Discussion

This paper introduces a battery-free, wireless-sensing system capable of continuously monitoring pressure and temperature at selected locations across the entire body, using sensors mounted at interfaces between the skin and a supporting mattress. The resulting information, simultaneously collected at multiple locations without irritation at the skin surface, offers potential to serve as the basis for early diagnosis and prevention of pressure injuries. The capabilities rely critically on two main advances over previously reported technologies: (i) a reliable, robust pressure sensor based on membrane deflection to meet all measurement requirements, including minimal hysteresis, absence of drift, high linearity, long-term stability and (ii) a battery-free, wireless-sensing platform and system that provides stable, and long-range communication capabilities for multiple mounting locations of interest. Benchtop studies, numerical simulations, clinical trials on hemiplegic and tetraplegic patients reveal all of the foundational and practical aspects of the technology.

Scaled clinical studies with these systems will help to define algorithms and thresholds for risk stratification, as described in Supplementary Note 8. The addition of other sensing modalities into these same device platforms could support further assessments of patient status, through measurements of full-body hemodynamics, galvanic skin responses, skin modulus values and other aspects and/or physiological signals. These directions appear promising for continued research in this area.

## Methods

### Fabrication of the battery-free, wireless electronic system

Fabrication began with patterning a flexible PCB substrate (Pyralux AP8535R, DuPont) to define electrical connections, vias, and the device outline with a direct UV (355-nm) laser ablation system (ProtoLaser U4, LPKF), followed by ultrasonic cleaning successively in oxide remover (Flux, Worthington Inc) for 2 min, deionized (DI) water for 2 min, and isopropyl alcohol (IPA, MG Chemicals) for 2 min to remove oxidation and organic residue. Electronic components included an NFC SoC (RF430FRL152H, Texas Instruments), an instrumentation amplifier (INA333, Texas Instruments), resistors, and capacitors, each placed using reflow soldering with low-temperature, solder paste (SMDLTLFP10T5, ChipQuik). The RF430FRL152H includes a NFC communication chip, a microcontroller and three 14-bit ADCs, with power delivery and wireless data communication from an NFC reader using ISO 15693 protocol at 13.56 MHz. The RF loop antenna operated at 13.56 MHz at a high-quality factor supported by low-loss tuning capacitors (GJM03-KIT-TTOL-DE, Murata Electronics). The pressure sensor completed a Wheatstone bridge circuit to convert its change in resistance to a change in voltage, passed to the instrumentation amplifier and delivered to an ADC of the NFC SoC. A NTC thermistor (NTCG064EF104FTBX, TDK Corporation) formed a voltage divider connected to another ADC of the NFC SoC to allow the change in temperature to be measured by a change in voltage from the ADC.

### Electromagnetic simulation for receiver coil

The commercial software ANSYS Electronics Desktop (HFFS) was used to perform electromagnetic (EM) finite-element analysis and determine the inductance *L*_*R*_, quality factor *Q*_R_, and impedance *Z*_11_, and matching capacitor *C*_R_ of the planar receiver coil. The coil diameter was 34.5 mm with five turns and the metal (copper) trace width, spacing, and thickness are 250, 100, and 18 μm, respectively. Lumped ports were used to the port impedance *Z*_11_ of the receiver coil. An adaptive mesh (tetrahedron elements) and a spherical radiation boundary (radius 500 mm) were adopted to ensure computational accuracy. *L* and *Q* were obtained as *L*_1_ = Im{*Z*_11_}/(2*π*f) = 1.96 μH and *Q*_1_ = |Im{*Z*_11_}/Re{*Z*_11_} |=43, where Re{*Z*_11_}, Im{*Z*_11_}, and f represent the real and imaginary parts of *Z*_11_, and the working frequency, respectively. The matching capacitor of the receiver coil at 13.56 MHz is *C*_R_ = 1*/*(2*π*f) Im{*Z*_11_} = 70 pF.

### NFC protocols, software control, and system operation

A RFID reader (TRF7970AEVM, Texas Instruments), connected to a computer/laptop, served as an interface to control the writing process in a NFC SoC mounted on a flexible PCB using a custom graphical user interface through ISO 15693. The NFC SoC integrated with the pressure sensor and the NTC thermistor supported data communication and wireless energy harvesting by the antenna reader (ID ISC. LRM2500-A, FEIG) with a transmission antenna that operates at 13.56 MHz, using ISOStart 2018 software for continuous, real-time data acquisition of ADC values from the NFC SoC in the protocol mode. As shown in Supplementary Fig. 26, for two multiplexed antennas (antenna 1 and antenna 2) with 10 wireless sensors, ISOStart2018 software in the protocol mode turns on antenna 1 for 2000 ms and initiates a sequential reading of ADC values of identified tags (wireless sensors) per 100 ms. After turning on antenna 2 for 2000 ms, sequential reads return the ADC values of each identified tag (wireless sensors) per 100 ms in the same manner. Software developed using Python enables classification and visualization of data for continuous, real-time monitoring.

### Fabrication of the tri-layered film

The process began with spin coating (1000 rpm for 60 s) and partially curing (100 °C for 2 min) a prepolymer of PDMS on a glass substrate followed by laminating a film of PI (75 μm in thickness) on top. Next, photolithography defined a pattern of resist (AZ nLOF 2035, MicroChem) to allow patterning of a bilayer of Cr/Au (10 nm/30 nm in thickness) deposited by electron beam evaporation *via* a liftoff process. Spin coating and curing (260 °C for 1 h) a film of PI (10 μm in thickness; PI-2545, HD Micro-Systems) yielded an encapsulation layer on top of the patterned Cr/Au. Forming mesh contact pads by RIE (O_2_, 100 mTorr, 100 W, 20 sccm, 20 min) and cutting the free-standing film with two opening cuts (2 × 0.25 mm^2^) to define the outline with a direct UV (355 nm) laser ablation system (ProtoLaser U4, LPKF) completed the formation of the tri-layer film.

### Assembly of the pressure sensor

The process began with preparation of piece of a Si wafer (8 × 8 × 0.5 mm^3^) with rounded edges and three opening cuts defined using a UV laser system (ProtoLaser U4, LPKF) and with a layer of Si_3_N_4_ formed by plasma enhanced chemical vapor deposition (PECVD; LpX CVD, STS). Next, electron beam evaporation of Cr/Au (10 nm/100 nm in thickness) through a shadow mask yielded patterns of electrical traces on the Si/Si_3_N_4_ substrate as a rigid substrate, followed by bonding a piece of a cover glass (3 × 4 × 0.15 mm^3^) using an epoxy resin (Loctite Epoxy Instant Mix 5 min, Loctite). The cover glass as a rigid sheet included an opening cut (2 × 2 mm^2^) in the middle region to allow both alignment and deflection of the tri-layered film and two opening cuts (0.7 × 0.7 mm^2^) for electrical connection using a UV laser system (ProtoLaser U4, LPKF). The tri-layered film (PI/Au/PI film with thicknesses of 75/0.03/10 μm; 3 × 4 × 0.075 mm^3^) with lithographically defined patterns of Au and two opening cuts (2 × 0.25 mm^2^) was bonded on the top surface of the rigid sheet, with electrical connections to the Cr/Au patterns on the rigid substrate formed using silver epoxy (8331-14G, MG chemicals). A soft, square pad of PDMS (1.5 × 1.5 × 0.265 mm^3^) was mounted on a suspended region between the opening cuts on the tri-layer film without chemical bonding to ensure conformal contact and stable operation under loading/unloading cycles. A rigid frame defined using a sheet of Si cut into a rectangular shape with rounded edges and an opening in the middle (7 × 7 mm^2^ inner lateral dimensions, 8 × 8 mm^2^ outer lateral dimensions and 500 μm in thickness) to protect the soft pad/tri-layered film/rigid sheet structure from mechanical/electrical damage by large shear stresses or excessive pressures was bonded on the rigid substrate followed by an epoxy-bonding of membrane film of PI (8 × 8 × 0.075 mm^3^) with two opening cuts (0.1 × 6 mm^2^). An elastomer (Dragon Skin, Smooth-On) patterned by a desktop cutting machine (Cameo 4, silhouette) or customized metallic punches served as a soft frame structure that allowed strong bonding on a membrane of PI and a rigid cover of Si using a surface treatment of (3-mercaptopropyl) trimethoxysilane (MPTMS; 175617, Sigma-Aldrich) and an epoxy resin. Supplementary Fig. 27 summarizes data on the maximum strength of adhesion of this soft frame (~2.63 N from a simple peel test, six times higher than values obtained without the surface chemical treatment). Also, a rigid, square pad of Si (3 × 3 × 0.5 mm^3^) was bonded on the center of the membrane of PI and a rigid cover of Si (8 × 8 × 5 mm^3^) using an epoxy resin to allow vertical displacements of the soft pad and tri-layered film in response to applied pressure, but prevents lateral deformations that could arise from shear loading. A layer of Si as the rigid cover rested on top to form a physical interface to the surroundings.

### Characterization of the pressure and temperature sensors

The setup for testing the pressure sensor (both wired and wireless) included a force gauge (Model M5-10, Mark-10) to measure the normal pressure, a digital multimeter (NI-USB 4065 Digital Multimeter) to measure the resistance of the sensor, and a motorized test stand (Mark-10, ESM303) to apply pressure with controlled loading and unloading rates. A dynamic mechanical tester (RSA-G2, TA Instruments) served as an alternative option for measuring the normal pressure and controlling the loading/unloading rates.

### Finite element analysis (FEA)

The commercial finite-element analysis (FEA) software ABAQUS was utilized to simulate and optimize the mechanical performance of the interconnect and the pressure sensor of the device. The objectives of the analysis were to ensure no plastic deformation in (1) the copper layer interconnects when the device undergoes different types of external loads (stretching, bending, and twisting) and (2) the gold layer inside the pressure sensor under the expected pressure range. The thin copper (18 μm thick) and gold (20 nm thick) layers were modeled by composite shell elements (S4R), and the other parts were modeled by hexahedron elements (C3D8R). The element size was tested to ensure the convergence and the accuracy of the simulation results. The elastic modulus (*E*) and Poisson’s ratio (*υ*) were *E*_PI_ = 3.2 GPa and *υ*_PI_ = 0.34 for PI; *E*_Cu_ = 119 GPa and *υ*_Cu_ = 0.34 for copper; *E*_Si_ = 130 GPa and *υ*_Si_ = 0.27 for silicon; *E*_PDMS_1_ = 1.6 MPa and *υ*_PDMS_1_ = 0.49 for PDMS 1 (encapsulation); *E*_PDMS_2_ = 0.5 MPa and *υ*_PDMS_2_ = 0.49 for PDMS 2 (soft pad).

### Electromagnetic simulations of the antenna system

FEA was used for electromagnetic simulations to determine the magnetic field distribution around the reader antennas at 13.56 MHz. The simulations used the commercial software ANSYS HFSS, in which tetrahedron elements were used in the solution with adaptive meshing convergence. An adaptive mesh convergence condition and a spherical radiation boundary (radius of 1000 mm) were adopted to ensure computational accuracy. The default material properties included in the HFSS material library were used in the simulation.

### Clinical trial protocol with hospitalized patients

The clinical study received an institutional review board (IRB) approval (2007 021 093) from Pusan National University Hospital. Volunteers that were recruited from the population of the study site (Kimhae Hansol Rehabilitation & Convalescent Hospital) joined the clinical trial after understanding the contents of the study and signing consent forms. The volunteers were classified into a healthy subject, a hemiplegic patient and a tetraplegic patient according to their symptoms in terms of disorder of perception or paralysis. The volunteers under the age of 18 or those with pressure injuries were excluded from the trials by considering the medical records of the volunteers, including age, gender, height, weight, body mass index (BMI), Braden scale score, presence of comorbidities such as hypertension and diabetes, paralysis, serum albumin level, and hemoglobin A1c level (normal: below 6.5%). The clinical trials began with cleaning the mounting locations of each subject by gently rubbing with alcohol wipes. Continuous measurements of pressure and temperature at skin interfaces against postural changes were evaluated on mounting locations of the patients prone to develop pressure injuries. The postural change of patients was captured with an infrared camera (Fortric 226, Fortric). All data recording occurred at 0.5–1 Hz (10–20 Hz for each sensor). Post-processing enabled data classification and visualization. After removing the sensors, skin condition at each mounting location was visually checked in consultation with doctors to identify any skin abnormalities. Also, feedback from patients and healthy subjects involved in trials is important in assessing acceptance and ease of use. After the clinical trials, doctors provided patients with a survey, the results of which appear in Supplementary Figs. 28–32.

## Supplementary information

Supplementary Information

## Data Availability

The data that support the findings of this study are available from the corresponding author upon reasonable request.
