# Author Correction: Battery-free, wireless soft sensors for continuous multi-site measurements of pressure and temperature from patients at risk for pressure injuries

**DOI:** 10.1038/s41467-021-26984-4

**Published:** 2021-11-18

**Authors:** Yong Suk Oh, Jae-Hwan Kim, Zhaoqian Xie, Seokjoo Cho, Hyeonseok Han, Sung Woo Jeon, Minsu Park, Myeong Namkoong, Raudel Avila, Zhen Song, Sung-Uk Lee, Kabseok Ko, Jungyup Lee, Je-Sang Lee, Weon Gi Min, Byeong-Ju Lee, Myungwoo Choi, Ha Uk Chung, Jongwon Kim, Mengdi Han, Jahyun Koo, Yeon Sik Choi, Sung Soo Kwak, Sung Bong Kim, Jeonghyun Kim, Jungil Choi, Chang-Mo Kang, Jong Uk Kim, Kyeongha Kwon, Sang Min Won, Janice Mihyun Baek, Yujin Lee, So Young Kim, Wei Lu, Abraham Vazquez-Guardado, Hyoyoung Jeong, Hanjun Ryu, Geumbee Lee, Kyuyoung Kim, Seunghwan Kim, Min Seong Kim, Jungrak Choi, Dong Yun Choi, Quansan Yang, Hangbo Zhao, Wubin Bai, Hokyung Jang, Yongjoon Yu, Jaeman Lim, Xu Guo, Bong Hoon Kim, Seokwoo Jeon, Charles Davies, Anthony Banks, Hyung Jin Sung, Yonggang Huang, Inkyu Park, John A. Rogers

**Affiliations:** 1 Center for Bio-Integrated Electronics, Northwestern University, Evanston, IL USA; 2 Department of Mechanical Engineering, Korea Advanced Institute of Science and Technology, Daejeon, Republic of Korea; 3 Department of Electrical and Computer Engineering, University of Illinois at Urbana–Champaign, Urbana, IL USA; 4 Department of Materials Science and Engineering, University of Illinois at Urbana Champaign, Urbana, IL USA; 5 Querrey Simpson Institute for Bioelectronics, Northwestern University, Evanston, IL USA; 6 State Key Laboratory of Structural Analysis for Industrial Equipment, Department of Engineering Mechanics, Dalian University of Technology, Dalian, People’s Republic of China; 7 Ningbo Institute of Dalian University of Technology, Ningbo, People’s Republic of China; 8 Department of Biomedical Engineering, Texas A&M University, College Station, TX USA; 9 Department of Mechanical Engineering, Northwestern University, Evanston, IL USA; 10 Advanced 3D Printing Technology Development Division, Korea Atomic Energy Research Institute, Daejeon, Republic of Korea; 11 Qualcomm Institute, La Jolla, CA USA; 12 Sibel Health Inc, Niles, IL USA; 13 Department of Rehabilitation Medicine, Gimhae Hansol Rehabilitation & Convalescent Hospital, Gimhae, Republic of Korea; 14 Department of Planning and Development, Gimhae Hansol Rehabilitation & Convalescent Hospital, Gimhae, Republic of Korea; 15 Department of Rehabilitation Medicine, Pusan national university hospital, Busan, Republic of Korea; 16 Department of Materials Science and Engineering, KAIST Institute for The Nanocentury (KINC), Korea Advanced Institute of Science and Technology, Daejeon, Republic of Korea; 17 Department of Materials Science and Engineering, Northwestern University, Evanston, IL USA; 18 Department of Mechanical Engineering, Kyung Hee University, Yongin, Republic of Korea; 19 Department of Biomedical Engineering, College of Future Technology, Peking University, Beijing, People’s Republic of China; 20 School of Biomedical Engineering, Korea University, Seoul, Republic of Korea; 21 Interdisciplinary Program in Precision Public Health, Korea University, Seoul, Republic of Korea; 22 Department of Electronic Convergence Engineering, Kwangwoon University, Seoul, Republic of Korea; 23 School of Mechanical Engineering, Kookmin University, Seoul, Republic of Korea; 24 Department of Electrical and Computer Engineering, Northwestern University, Evanston, IL USA; 25 School of Chemical Engineering, Sungkyunkwan University, Suwon, Republic of Korea; 26 School of Electrical Engineering, Korea Advanced Institute of Science and Technology, Daejeon, Republic of Korea; 27 Department of Electrical and Computer Engineering, Sungkyunkwan University, Suwon, Republic of Korea; 28 Biomedical Manufacturing Technology Center, Korea Institute of Industrial Technology (KITECH), Yeongcheon, Republic of Korea; 29 Department of Aerospace and Mechanical Engineering, University of Southern California, Los Angeles, CA USA; 30 Department of Applied Physical Sciences, University of North Carolina at Chapel Hill, Chapel Hill, NC USA; 31 Department of Electrical and Computer Engineering, University of Wisconsin-Madison, Madison, WI USA; 32 NeuroLux, Inc, Glenview, IL USA; 33 Department of Organic Materials and Fiber Engineering, Soongsil University, Seoul, Republic of Korea; 34 Carle Neuroscience Institute, Carle, Physician Group, Urbana, IL USA; 35 Departments of Civil and Environmental Engineering, Northwestern University, Evanston, IL USA; 36 Department of Biomedical Engineering, Northwestern University, Evanston, IL USA; 37 Department of Neurological Surgery, Feinberg School of Medicine, Northwestern University, Chicago, IL USA

**Keywords:** Lifestyle modification, Biomedical engineering, Electrical and electronic engineering, Mechanical engineering, Electronic devices

Correction to: *Nature Communications* 10.1038/s41467-021-25324-w, published online 24 August 2021.

In the Acknowledgements section of this article the grant number relating to Korea Government (MSIT) given for Yong Suk Oh, Seokjoo Cho, Hyeonseok Han, Kyuyoung Kim, Seunghwan Kim, Min Seong Kim, Jungrak Choi, and Inkyu Park was incorrectly given as 2018R1A2B200491013 and should have been 2021R1A2C3008742. The original article has been corrected.

