# Supplementary material for: Battery-free, wireless soft sensors for continuous multi-site measurements of pressure and temperature from patients at risk for pressure injuries
Source: Nat Commun. 2021 Aug 24;12:5008. doi: 10.1038/s41467-021-25324-w (PMC8385057; doi:10.1038/s41467-021-25324-w)
Supplement: Supplementary file 1 — Supplementary Information [file 41467_2021_25324_MOESM1_ESM.pdf]

Supplementary Information

Battery-Free, Wireless Soft Sensors for Continuous Multi-Site Measurements of Pressure and Temperature for Patients at Risk for Pressure Injuries

Yong Suk Oh[1,2†], Jae Hwan Kim[3,4,5†], Zhaoqian Xie[6,7†], Seokjoo Cho[2], Hyeonseok Han[2], Sung Woo Jeon[3], Minsu Park[5], Myeong Namkoong[8], Raudel Avila[9], Zhen Song[6,7], Sung-Uk Lee[10], Kabseok Ko[11], Jungyup Lee[12], Je-Sang Lee[13], Weon Gi Min[14], Byeong-Ju Lee[15], Myungwoo Choi[16], Ha Uk Chung[12], Jongwon Kim[12,17, 18], Mengdi Han[19], Jahyun Koo[20,21], Yeon Sik Choi[1, 5,17], Sung Soo Kwak[5], Sung Bong Kim[4,5], Jeonghyun Kim[22], Jungil Choi[23], Chang-Mo Kang[24], Jong Uk Kim[5,25], Kyeongha Kwon[26], Sang Min Won[27], Janice Mihyun Baek[4], Yujin Lee[4], So Young Kim[4], Wei Lu[1], Abraham Vazquez-Guardado[1,5], Hyoyoung Jeong[5], Hanjun Ryu[1], Geumbee Lee[1,5,17], Kyuyoung Kim[2], Seunghwan Kim[2], Min Seong Kim[2], Jungrak Choi[2], Dong Yun Choi[28], Quansan Yang[5,17], Hangbo Zhao[29], Wubin Bai[30], Hokyung Jang[31], Yongjoon Yu[32], Jaeman Lim[5], Xu Guo[6,7], Bong Hoon Kim[33], Seokwoo Jeon[16], Charles Davies[34], Anthony Banks[1,5], Hyung Jin Sung[2], Yonggang Huang[1,9,17,35]*, Inkyu Park[2]*, John A. Rogers[1,5,16,17,36,37]*

[1]Center for Bio-Integrated Electronics, Northwestern University, Evanston, IL 60208, USA. [2]Department of Mechanical Engineering, Korea Advanced Institute of Science and Technology, Daejeon 34141, Republic of Korea. [3]Department of Electrical and Computer Engineering, University of Illinois at Urbana–Champaign Urbana, Champaign, IL 61801, USA. [4]Department of Materials Science and Engineering, University of Illinois at Urbana Champaign, Urbana, IL 61801, USA. [5]Querrey Simpson Institute for Bioelectronics, Northwestern University, Evanston, IL 60208, USA. [6]State Key Laboratory of Structural Analysis for Industrial Equipment, Department of Engineering Mechanics, Dalian University of Technology, Dalian 116023, P.R.China. [7]Ningbo Institute of Dalian University of Technology, Ningbo 315016, P.R.China. [8]Department of Biomedical Engineering, Texas A&M University, College Station, TX 77843, USA. [9]Department of Mechanical Engineering, Northwestern University, Evanston, IL 60208, USA. [10]Advanced 3D Printing Technology Development Division, Korea Atomic Energy Research Institute, Daejeon 34057, Republic of Korea. [11]Qualcomm Institute, La Jolla, CA 92093, USA. [12]Sibel Health Inc, Niles, IL 60714, USA. [13]Department of Rehabilitation Medicine, Gimhae Hansol Rehabilitation & Convalescent Hospital, Gimhae 50924, Republic of Korea. [14]Department of planning and development, Gimhae Hansol Rehabilitation & Convalescent Hospital, Gimhae 50924, Republic of Korea. [15]Department of rehabilitation medicine, Pusan national university hospital, Busan 49241, Republic of Korea. [16]Department of Materials Science and Engineering, KAIST Institute for The Nanocentury (KINC), Korea Advanced Institute of Science and Technology, Daejeon 34141, Republic of Korea. [17]Department of Materials Science and

Engineering, Northwestern University, Evanston, IL 60208, USA. [18]Department of Mechanical Engineering, Kyung Hee University, Yongin 17104, Republic of Korea. [19]Department of Biomedical Engineering, College of Future Technology, Peking University, Beijing 100871, P. R. China. [20]School of Biomedical Engineering, Korea University, Seoul 02841, Republic of Korea. [21]Interdisciplinary Program in Precision Public Health, Korea University, Seoul 02841, Republic of Korea. [22]Department of Electronic Convergence Engineering, Kwangwoon University, Seoul 01897, Republic of Korea. [23]School of Mechanical Engineering, Kookmin University, Seoul 02707, Republic of Korea. [24]Department of Electrical and Computer Engineering, Northwestern University, Evanston, IL 60208, USA. [25]School of Chemical Engineering, Sungkyunkwan University, Suwon 16419, Republic of Korea. [26]School of Electrical Engineering, Korea Advanced Institute of Science and Technology, Daejeon 34141, Republic of Korea. [27]Department of Electrical and Computer Engineering, Sungkyunkwan University, Suwon 16419, Republic of Korea. [28]Biomedical Manufacturing Technology Center, Korea Institute of Industrial Technology (KITECH), Yeongcheon 38822, Republic of Korea. [29]Department of Aerospace and Mechanical Engineering, University of Southern California, Los Angeles, CA 90089, USA. [30]Department of Applied Physical Sciences, University of North Carolina at Chapel Hill, Chapel Hill, NC 27514, USA. [31]Department of Electrical and Computer Engineering, University of Wisconsin-Madison, Madison, WI 53706, USA. [32]NeuroLux, Inc., Glenview, IL 60093, USA. [33]Department of Organic Materials and Fiber Engineering, Soongsil University, Seoul 06978, Republic of Korea. [34]Carle Neuroscience Institute, Carle, Physician Group, Urbana, IL 61801, USA. [35]Departments of Civil and Environmental Engineering, Northwestern University, Evanston, IL 60208, USA. [36]Department of Biomedical Engineering, Northwestern University, Evanston, IL 60208, USA. [37]Department of Neurological Surgery, Feinberg School of Medicine, Northwestern University, Chicago, IL 60611, USA.

Table of Contents

Supplementary Note 1. Comparison with previous research
Supplementary Note 2. Upper and lower bounds of pressure sensor
Supplementary Note 3. Repeatability, sensitivity and reproducibility
Supplementary Note 4. Calibration process of wireless sensors
Supplementary Note 5. Shear force-related pressure injuries
Supplementary Note 6. Discontinuous periods of data collection in clinical trials
Supplementary Note 7. Effective area of pressure sensor in clinical trials
Supplementary Note 8. Threshold of pressure injury
Supplementary Fig. 1. Circuit diagram of the device.
Supplementary Fig. 2. Mechanical reliability of the battery-free, wireless sensing platform.
Supplementary Fig. 3. Procedure for fabricating the pressure sensor with temperature sensor.
Supplementary Fig. 4. Robustness of the pressure sensor against shear stress.
Supplementary Fig. 5. Experimental setup for evaluating the characteristics of the pressure sensor.
Supplementary Fig. 6. Average responses of 3 pressure sensors to applied loads of 6 kPa at different initial resistances of 10.2 kΩ and 20.1 kΩ, respectively.
Supplementary Fig. 7. Sensitivity of a pressure sensor over a relevant range of pressures within the upper and lower bounds of fractional changes in resistance.
Supplementary Fig. 8. Effective modulus of a pressure transducer with a thickness of 2 mm under compressive loading.
Supplementary Fig. 9. Average responses of 10 pressure sensors to applied loadings of 3 kPa and 6 kPa.
Supplementary Fig. 10. Temperature dependence of the pressure sensor.
Supplementary Fig. 11. Experimental setup for evaluating the response of a pressure sensor at different interfacial substrates.
Supplementary Fig. 12. Experimental setup for evaluating the response of a pressure sensor for the case of substrates with different radii of curvature.
Supplementary Fig. 13. Fractional change in resistance of the sensor at different values of RH.
Supplementary Fig. 14. Fractional change in resistance at RH of 80% for 12 hours.
Supplementary Fig. 15. Comparative studies of pressure measurements on a human subject.
Supplementary Fig. 16. Stability of the response of the pressure sensor during various deformations.
Supplementary Fig. 17. Magnetic field strength along the central axis of the antenna as a function of distance out of the plane of the antenna coil for three different RF powers.
Supplementary Fig. 18. Block diagram of the system.
Supplementary Fig. 19. Evaluation of the receiver antenna of the wireless sensing platform, to

determine the center frequency and the Q factor.

Supplementary Fig. 20. Electrical stability of the battery-free, wireless sensing platform.

Supplementary Fig. 21. Change in the ADC value from the NFC SoC for a wireless device operating while completely submerged in water for 60 min.

Supplementary Fig. 22. Continuous measurements of pressure and temperature from a healthy subject (30-year-old male, 72 kg, 180 cm) using 4 wireless sensing platforms placed in proxi mity to one another.

Supplementary Fig. 23. Photographic image of changes in posture of the subject with right hemiplegia.

Supplementary Fig. 24. Continuous measurements of pressure and temperature from a subject with hemiplegia and stroke (61-year-old male, 57 kg, 170 cm) using the wireless sensing platform during sleep time (overnight).

Supplementary Fig. 25. Photographic images of skin in the region of the right elbow and the heel after removing the wireless devices.

Supplementary Fig. 26. Programming of ISOStart2018 software in protocol mode for operation of multiplexed antenna and multiple sensors.

Supplementary Fig. 27. Comparison of adhesive strength between the MPTMS-treated silicone (Dragon Skin) and the PI film for epoxy bonding.

Supplementary Fig. 28. Feedback from the healthy subject (30-year-old male, 72 kg, 180 cm) in Fig. 5.

Supplementary Fig. 29. Feedback from the healthy subject (30-year-old male, 72 kg, 180 cm) in Fig. 6.

Supplementary Fig. 30. Feedback from the patient with right hemiplegia (47-year-old female, 62 kg, 160 cm) in Fig. 7.

Supplementary Fig. 31. Feedback from the patient with general paralysis (83-year-old male, 40 kg, 150 cm) in Fig. 8.

Supplementary Fig. 32. Feedback from the patient with hemiplegia and stroke (61-year-old male, 57 kg, 170 cm) in Supplementary Fig. 24.

Supplementary Table 1. Comparison for battery-free, wireless sensors.

Supplementary References

Supplementary Note 1. Comparison with previous research

In a previous study[1], the pressure sensor incorporated a spiral-shaped, thin, monocrystalline membrane of Si (a thickness of 200 nm; E of 130 GPa), a polyethylene terephthalate substrate (PET; a thickness of 5 µm; E of ~4.5 GPa) and an encapsulating layer of PDMS (a thickness of 350 µm; E of ~1MPa). The sensing mechanism depends on the Poisson effect associated with the encapsulating PDMS layer and the consequent stretching of the spiral-shaped Si. The PET film ensures a uniform strain distribution and improves the mechanical robustness of the spiral-shaped Si structure. These same effects, however, decrease the sensitivity (< 0.002 kPa^{-1}, a fractional change of less than 2% in resistance under 10 kPa) to values much lower than those that could be supported by the Si membrane and its high gauge factor (~50). Fabricating/transferring the thin spiral-shaped, p-doped, Si membrane on PET film without damage includes a series of complex processes, resulting in reduced device reproducibility.

By contrast, the pressure sensor in the present study is much different in its materials, designs and operating principles. Specifically, the device includes an Au trace in a tri-layered film as a sensing element, with a combined design that incorporates both rigid and soft components, as shown in Figure. 2a and Supplementary Fig. 3. The sensing mechanism of the device depends on deflection-induced tensile strain under applied pressures. As illustrated in Figure 2c, d, a well-defined area supports a patterned Au trace in the tri-layered film. A soft frame on a membrane of PI functions to control the sensitivity over relevant pressure ranges (<0.0006, a fractional change of less than 0.6% in resistance under 10 kPa). Supplementary Fig. 9 shows average responses of 10 pressure sensors to applied loads of 3 kPa and 6 kPa, respectively. A rigid frame and rigid pad protect the sensing components from mechanical/electrical damage that could otherwise result from shear stresses or excessive pressures, in a manner that does not decrease the sensitivity, as shown in Supplementary Fig. 4. Also, the pressure sensor shows long-term stability and mechanical durability under 10,000 repeated loadings of 4 kPa, as shown in Figure. 2g. Additional measurements demonstrate the level of repeatability in the responses across various batches of devices, as highlighted in Supplementary Fig. 9.

Table. S1 summarizes the current design and the previous design in terms of pressure sensor and wireless platform; it also compares the clinical trials. Many important details are different in the current system, and the cumulative consequences of these advances serve as the basis for extensive, successful demonstrations of the technology with actual patients in operating hospitals.

In the previous design, the pressure/temperature sensor locates inside the coil of the receiver antenna with an outer diameter of 16 mm, suitable for mounting on the skin directly at the locations of the protruding regions of highest risk (e.g., elbow and toe). This small-coil design limits the communication range and its mounting location also leads to concerns about unwanted perturbations at the skin interface. By contrast, the current design exploits a separate coil for a receiver antenna that has an outer

diameter of 34.5 mm, connected by deformable serpentine traces to the pressure/temperature sensors. This separated layout allows for comparatively large coils and corresponding increases in the working distance. The separate, small temperature/pressure sensor module (<10 mm × 10 mm) is easy to attach to the regions of skin of interest. This scheme also enables stable detection during changes in position or posture. Supplementary note (1) and Table S1 highlight these issues.

Supplementary Note 2. Upper and lower bounds of pressure sensor

The pressure sensor, including the Au trace with a yield strain of 0.3%, has a maximum fractional change in resistance of 0.6% under loading as an upper bound and a fractional change of 0 % under unloading as a lower bound. The soft frame in Figure 2a and Supplementary Fig. 3 functions to modulate the sensitivity over the operating ranges of pressure, related to the sensitivity within the upper bound. In Fig.2d, the pressure sensors show a sensitivity of 0.00053 kPa^{-1} without the soft frame, 0.0003 kPa^{-1} with a soft frame (E = 100 kPa) and 0.0001 kPa^{-1} with an extremely soft frame (E = 100 kPa), respectively. These results show that the pressure sensors can provide maximum fractional change in resistance of 0.6% with negligible hysteresis and drift in the pressure range of 0-11.4 kPa, 0-21.0 kPa and 0-58.2 kPa in Supplementary Fig. 8. Also, structural designs of device for the ratio of width/height of soft frame or a distance of metal trace from the neutral plane supports modulating the sensitivity of pressure sensor over relevant pressure ranges within the maximum fractional change of resistance of 0.6%.

Supplementary Note 3. Repeatability, sensitivity and reproducibility

The battery-free, wireless pressure sensors show good repeatability and reproducibility. Supplementary Fig. 9 shows average responses of 10 pressure sensors to applied loads of 3 kPa and 6 kPa. The effective area of the patterned Au traces (a width of 7μm) in the tri-layered film, rather than the original resistance, is important for realizing similar sensitivity, as shown in Fig. 2b, c. The sensitivity of the wireless pressure sensor depends on the combination of resistances in the Wheatstone bridge and the resistance that sets the gain in the instrumentation amplifier. Each wireless sensor requires a calibration process, performed before clinical trials to ensure proper, accurate operation.

Supplementary Note 4. Calibration process

In the calibration process, fractional changes in resistance ($\Delta R/R$) measured from the sensor converts to pressure values. The initial resistance does not affect the response to pressure. Integration of the pressure sensor into a wireless platform completes a Wheatstone bridge circuit to convert the fractional change in resistance to a change in voltage, passed to the instrumentation amplifier and delivered to ADC values

of the NFC SoC. Both the initial voltage and voltage change of the sensor depend on the combination of resistances in the Wheatstone bridge and the resistance that sets the gain in the instrumentation amplifier. For this reason, the calibration process for each sensor uses the change of the ADC value (voltage), not the fractional change in voltage. Also, each device requires a one-point calibration using the relationship between pressure and voltage, as shown in Figure 4a. The result provides accurate, continuous conversion from the change of the ADC value to the pressure due to high linearity and minimum hysteresis.

On the other hand, the temperature sensor (NTCG064EF104FTBX, TDK Corporation) has the following relationship (1) between temperature (T) and resistance (R_{NTC}) provided by data sheet. The temperature sensor forms a voltage divider circuit to convert R_{NTC} to V_{out} using Equation (2), delivered to an ADC of the NFC SoC. The relationship (3) between T and V_{out}, derived from Equation (1) and (2), supports accurate, continuous conversion of ADC values collected from the wireless temperature sensor.

$$R_{\mathrm{NTC}} = R_{\mathrm{T0}} \exp \varphi \left(\frac{1}{T+273} - \frac{1}{T_0 + 273} \right) \qquad (1)$$

where R_{T0} is a reference resistance of 100 kΩ, φ is a correcting factor of 4250 and T_0 is a reference temperature of 25 ºC, respectively.

$$V_{\mathrm{out}} = V_{\mathrm{in}} \left(\frac{R_{\mathrm{T1}}}{R_{\mathrm{NTC}} + R_{\mathrm{T1}}} \right) \qquad (2)$$

where R_{T1} is a divided resistance of 50 kΩ and V_{in} is an applied voltage of 1.5V, respectively.

$$T = \left[\frac{1}{\varphi} \ln \left(\frac{R_{\mathrm{T1}}}{R_{\mathrm{T0}}} \left(\frac{V_{in}}{V_{out}} - 1 \right) \right) + \frac{1}{(T_0 + 273)} \right]^{-1} - 273 \qquad (3)$$

where R_{T0} is a reference resistance of 100 kΩ, R_{T1} is a divided resistance of 50 kΩ, φ is a correcting factor of 4250, T_0 is a reference temperature of 25 ºC, V_{in} is an applied voltage of 1.5 V, respectively.

Supplementary Note 5. Shear force-related pressure injuries

Pressure is a common type of force generated by body weight on the skin and underlying soft tissue, compared to shear force for most patients.[2] Prolonged pressure applied directly to the skin and underlying soft tissue increases capillary occlusion, leading to tissue ischemia with associated localized hypoxia.[3] This process can impair the delivery of vital nutrients and oxygen to the local environment of the cells, resulting in the local breakdown of the soft tissues. Especially, the effect of pressure on

developing the skin injuries is dominant for patients with perception disorders (e.g., a hemiplegic patient or a tetraplegic patient) who are confined to a bed. Shear force can be important in specific cases, including a semi-sitting position in a bed or a sitting position in a wheelchair (e.g., a paraplegic patient).[4] The effects of shear force on these injuries are associated with a reclining angle of the back support.

Supplementary Note 6. Discontinuous periods of data collection in clinical trials

These events have two causes. First, large tilt angles (greater than ~60°) of the receiver coil relative to the primary antenna can follow from changes in posture, thereby reducing the working distance and, in some cases, causing discontinuities in the data. This issue can be minimized through shaped designs of the antenna coil (e.g., an elliptical shape) and double-loop layouts for the primary antenna, as described in the use of NFC interfaces in other contexts[8]. Second, movement of the receiver antenna out of the region encompassed by the primary antenna can lead to similar effects, also due to changes in posture and position. The two multiplexed, commercial antennas (800 mm × 600 mm) in this study provide a stable magnetic field distribution over the full area of the body, but not over the full area of the clinical bed (2000 mm × 800 mm). This issue can be resolved with antennas sufficiently large for covering the entire size of clinical bed.

Supplementary Note 7. Effective area of pressure sensor in clinical trials

In general, the specific area applied by the weight of human body is smaller or larger than the interfacial area of a single sensor. This mismatch could hinder measurement of accurate, reliable pressure values on a location of interest. The strategy for resolving this issue involves an array of pressure sensors[5,6] or a collection of multiple devices in proximity to one another[7]. The array enables mapping of pressure with a relatively high resolution over regular spatial intervals across a specific area. The platform for generating an array of pressure sensor requires additional components (e.g., multiplexer and controller) on the NFC SoC, to support data communication with 8, 16 or 32 channels of ADC depending on the specifications of the multiplexer, in a sequential mode. Placing multiple devices in proximity to one another provides another option, depending on critical sites of a patient's body. Supplementary Fig. 22a shows photographs of a subject with 4 devices mounted near the sacrum in a prone position.

Supplementary Note 8. Threshold of pressure injury

The threshold associated with pressure magnitudes and durations depends on body locations and the health condition of a patients, including issue related to sensory perception, activity, mobility, nutrition and chronic disease state. These factors[3,9–12] create challenges in defining accurate threshold values for pressure injuries. For this reason, the National Pressure Injury Advisory Panel (NPIAP) provides

guidelines for evidence-based recommendations to prevent and treat pressure injuries that could be used by health professionals throughout the world. A repositioning (e.g., prone, side-lying or supine) of a patient lying in bed at regular intervals reduces the development of pressure injuries. The Braden scale serves as a tool to assess the level of risk for pressure injuries, by providing medical teams or nurses with quantitative scores to categorize high-risk patients. In this context, advanced technologies for continuous multi-site measurements of pressure and temperature at skin interfaces have potential to provide supplementary information, beyond simple assessments of time duration, as an alarm for identifying risk. As outlined in the discussion section, statistical, scaled clinical studies performed with the technology introduced here will be helpful for defining algorithms and thresholds for risk stratification of subjects according to body type, age, condition, body location and other key factors. We envision a deployment strategy that would begin with a basic use of data from the sensors to guide clinical care decisions where, over time, increasing amounts of data and correlated conditions will enable more sophisticated uses of the information.

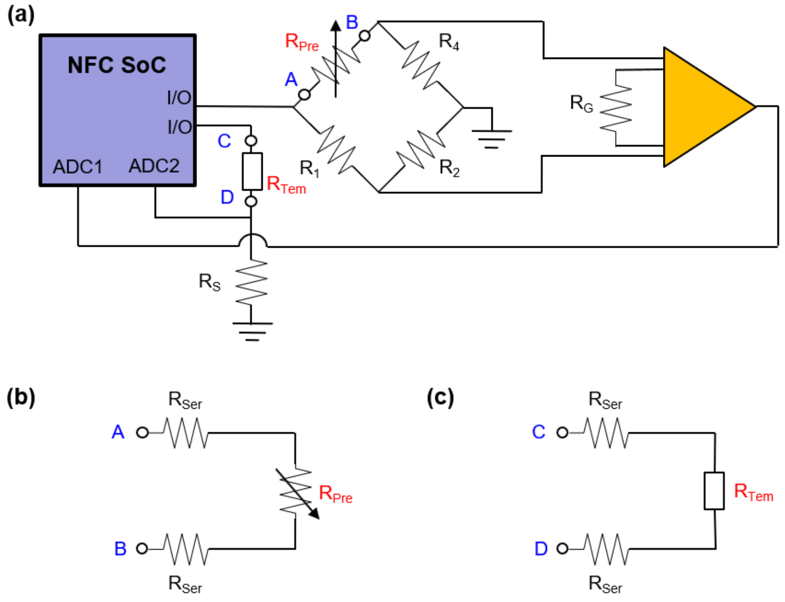

Supplementary Fig. 1. Circuit diagram of the device. (a) Circuit diagram of the wireless sensing platform integrated with pressure sensor connected to Wheatstone bridge and temperature sensor connected to voltage divider. (b) Circuit diagram of pressure sensor connected to serpentine traces. (c) Circuit diagram of the temperature sensor connected to serpentine traces.

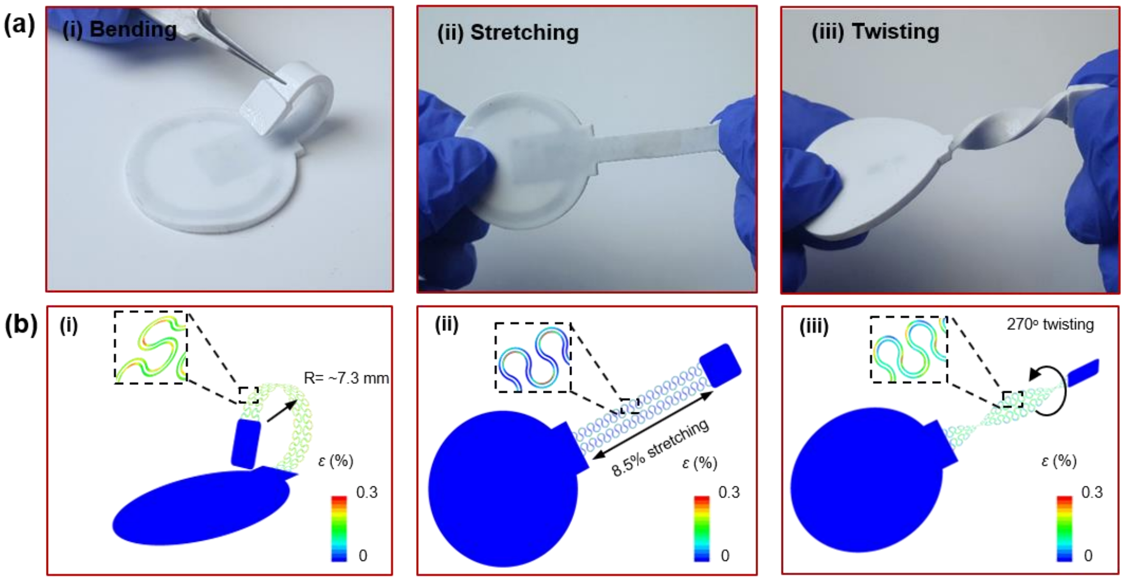

Supplementary Fig. 2. Mechanical reliability of the battery-free, wireless sensing platform. (a) Photographic images of the device in (i) bent, (ii) stretched, (iii) twisted configurations. (b) Finite element modeling of the mechanics for these three cases.

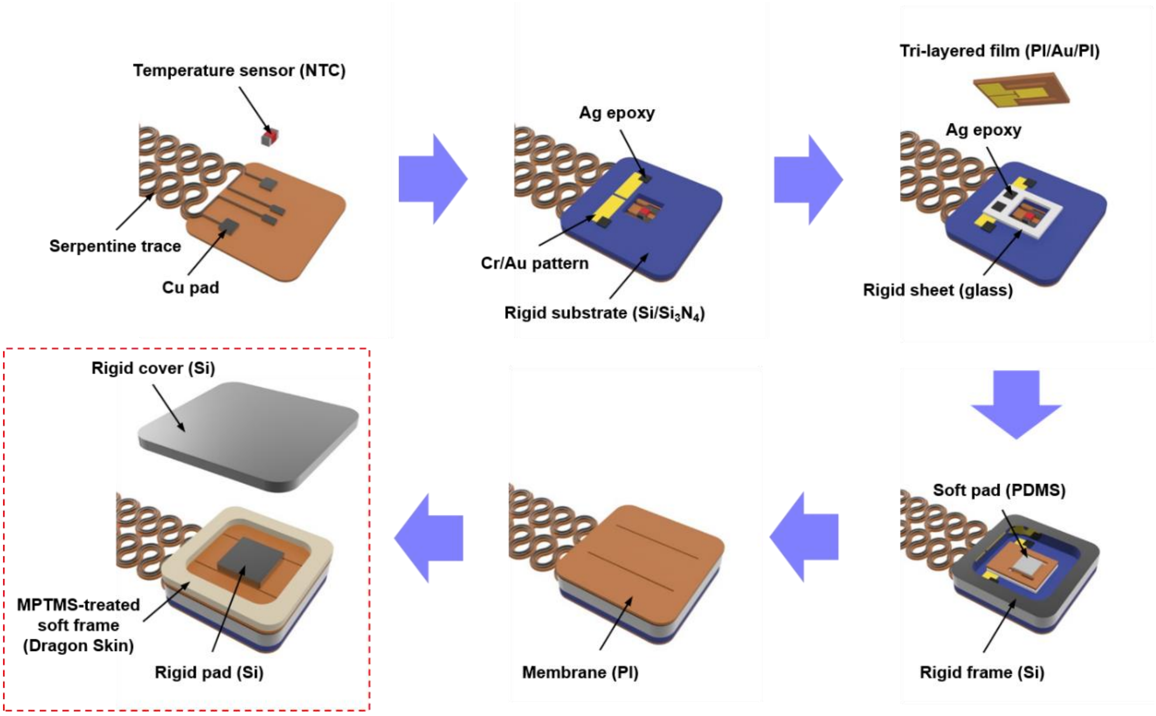

Supplementary Fig. 3. Procedure for fabricating the pressure sensor with temperature sensor.

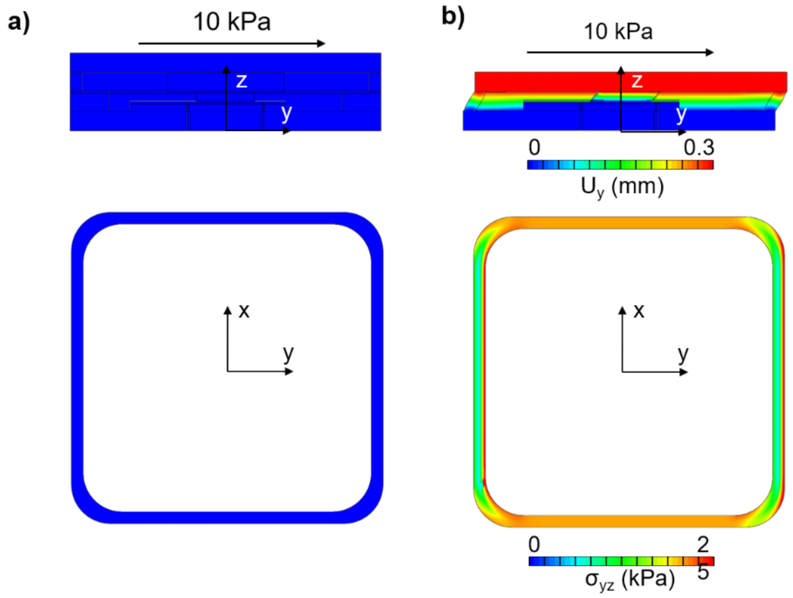

Supplementary Fig. 4. Robustness of the pressure sensor against shear stress. Modeling results for the deformation and the shear stress σ_{yz} on the upper surface of the dragon skin for (a) design A of the pressure sensor with two-stage structure and (b) design B of the pressure sensor with one-stage structure under the shear stress of 10 kPa. U_y and σ_{yz} represent the displacement in the y direction and the y-z shear stress, respectively.

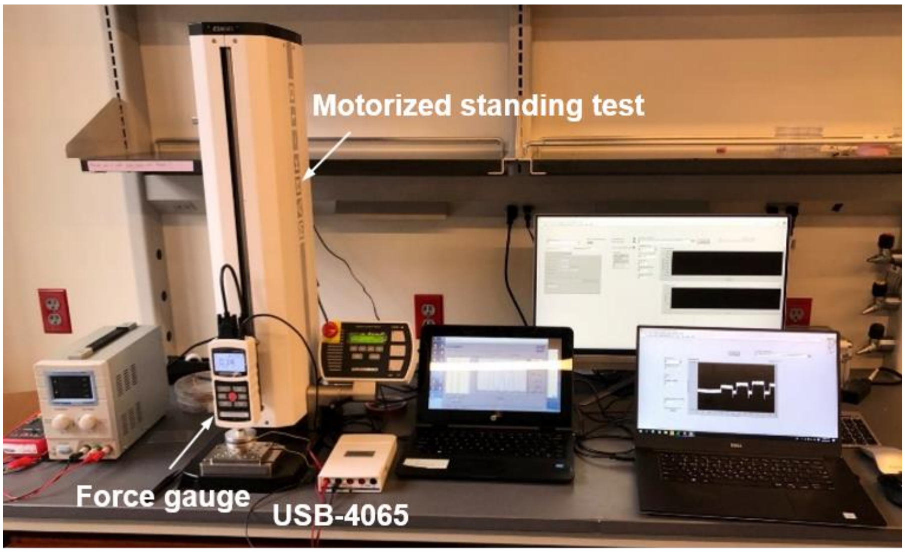

Supplementary Fig. 5. Experimental setup for evaluating the characteristics of the pressure sensor. The setup includes a digital multimeter (USB 4065, NI) and a force gauge (Mark-10) equipped with motorized standing test (ESM303).

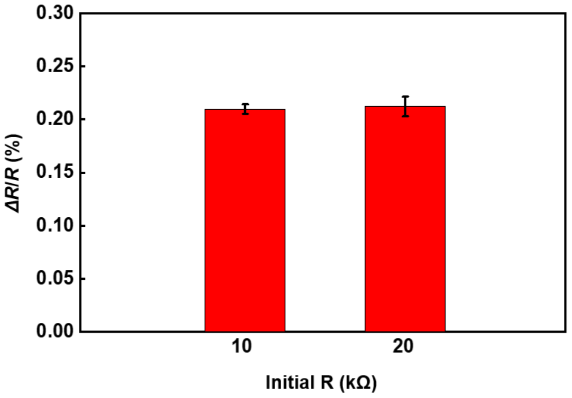

Supplementary Fig. 6. Average responses of 3 pressure sensors to applied loads of 6 kPa at different initial resistances of 10.2 kΩ and 20.1 kΩ, respectively.

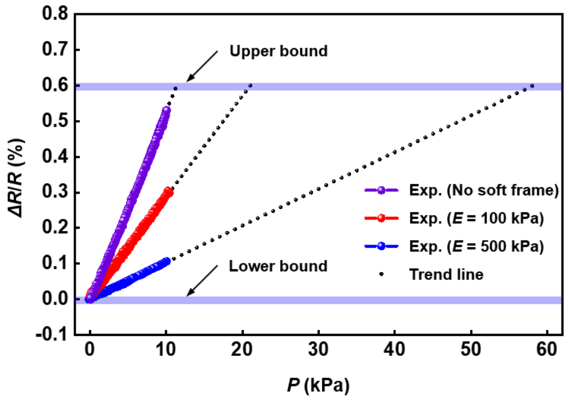

Supplementary Fig. 7. Sensitivity of a pressure sensor over a relevant range of pressures within the upper and lower bounds of fractional changes in resistance.

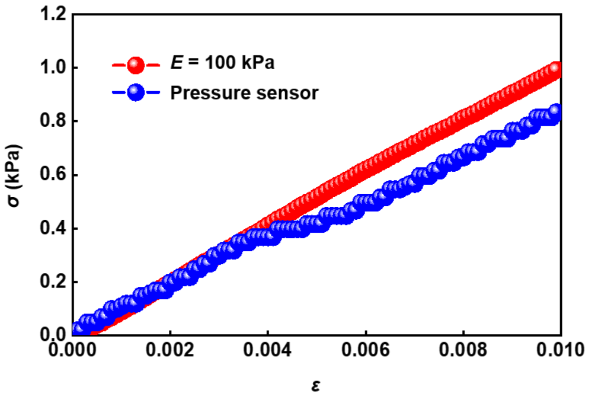

Supplementary Fig. 8. Effective modulus of a pressure transducer with a thickness of 2 mm under compressive loading.

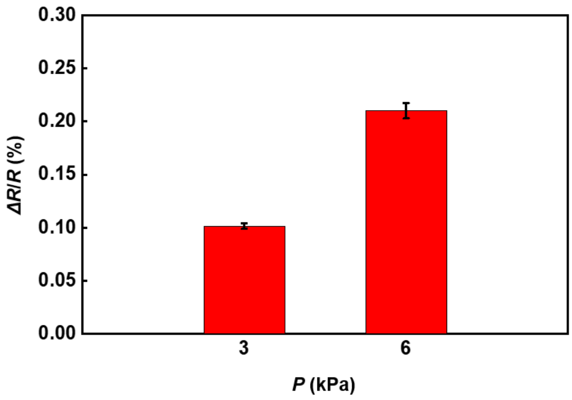

Supplementary Fig. 9. Average responses of 10 pressure sensors to applied loadings of 3 kPa and 6 kPa.

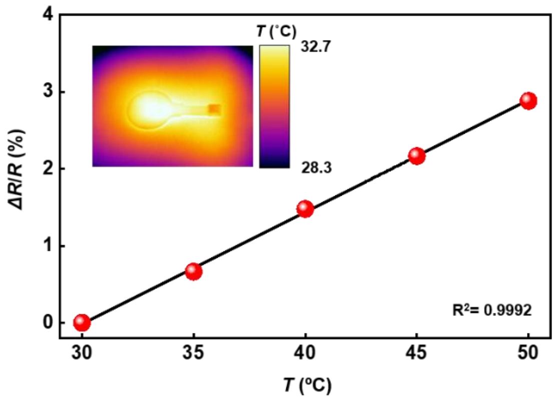

Supplementary Fig. 10. Temperature dependence of the pressure sensor.

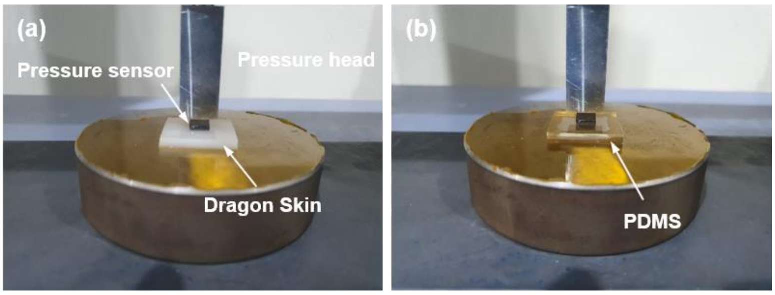

Supplementary Fig. 11. Experimental setup for evaluating the response of a pressure sensor at different interfacial substrates. Photographic images of (a) Dragon skin with *E* = 0.1 MPa and (b) PDMS with *E* = 1 MPa.

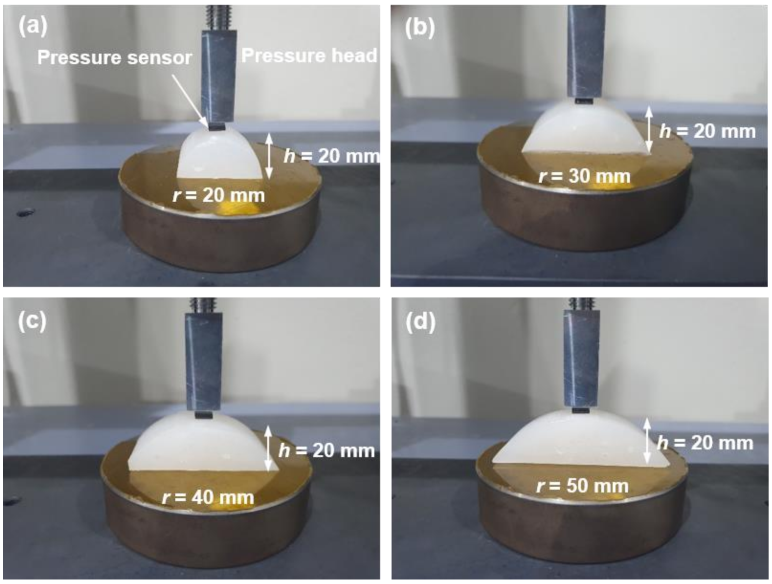

Supplementary Fig. 12. Experimental setup for evaluating the response of a pressure sensor for the case of substrates with different radii of curvature. (a-d) Photographic images for cases with different radii of curvature (Dragon Skin with E = 100 kPa).

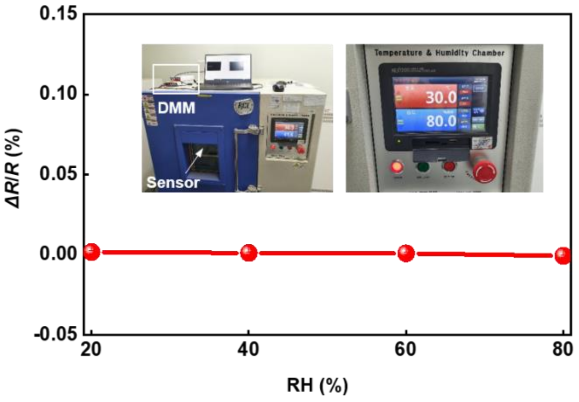

Supplementary Fig. 13. Fractional change in resistance of the sensor at different values of RH.

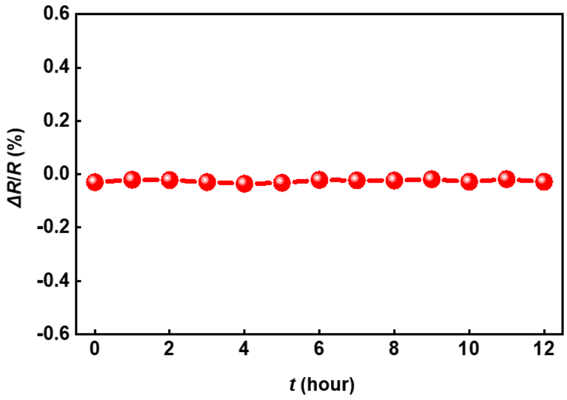

Supplementary Fig. 14. Fractional change in resistance at RH of 80% for 12 hours.

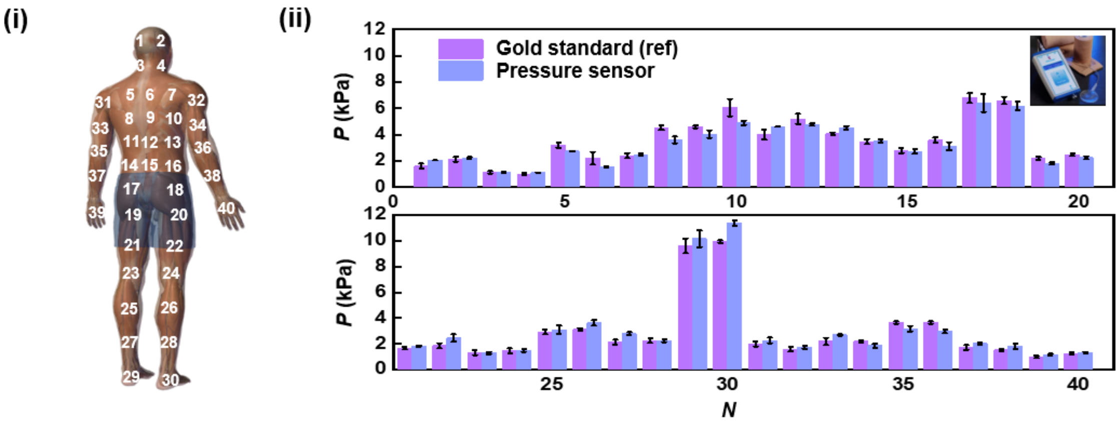

Supplementary Fig. 15. Comparative studies of pressure measurements on a human subject. (i) Schematic illustration of different anatomical locations for measuring the pressure at the skin-mattress interface. (ii) Feasibility evaluation of the pressure sensor on each mounting locations of the subject lying on bed. A commercial pressure sensor, Picopress, was used as a reference.

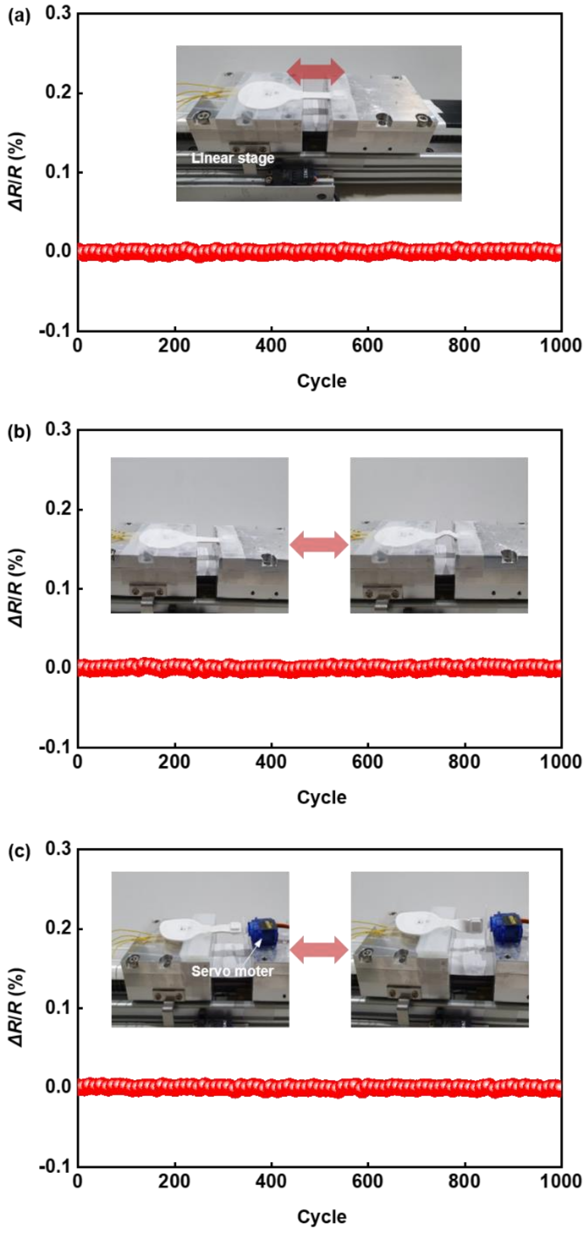

Supplementary Fig. 16. Stability of the response of the pressure sensor during various deformations. (a-c) Fractional change in resistance of the piezoresistive strain gauge during 1,000 repeated cycles of stretching (8%), bending (7mm) and twisting (180°), respectively.

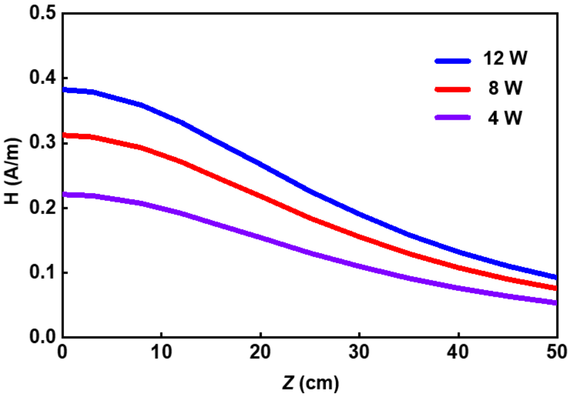

Supplementary Fig. 17. Magnetic field strength along the central axis of the antenna as a function of distance out of the plane of the antenna coil for three different RF powers.

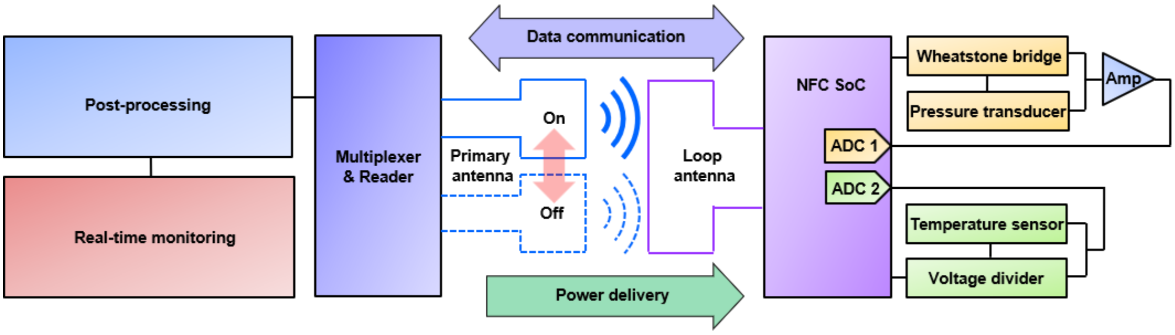

Supplementary Fig. 18. Block diagram of the system.

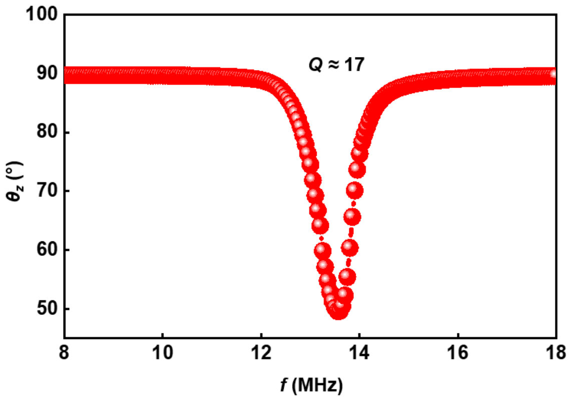

Supplementary Fig. 19. Evaluation of the receiver antenna of the wireless sensing platform, to determine the center frequency and the Q factor.

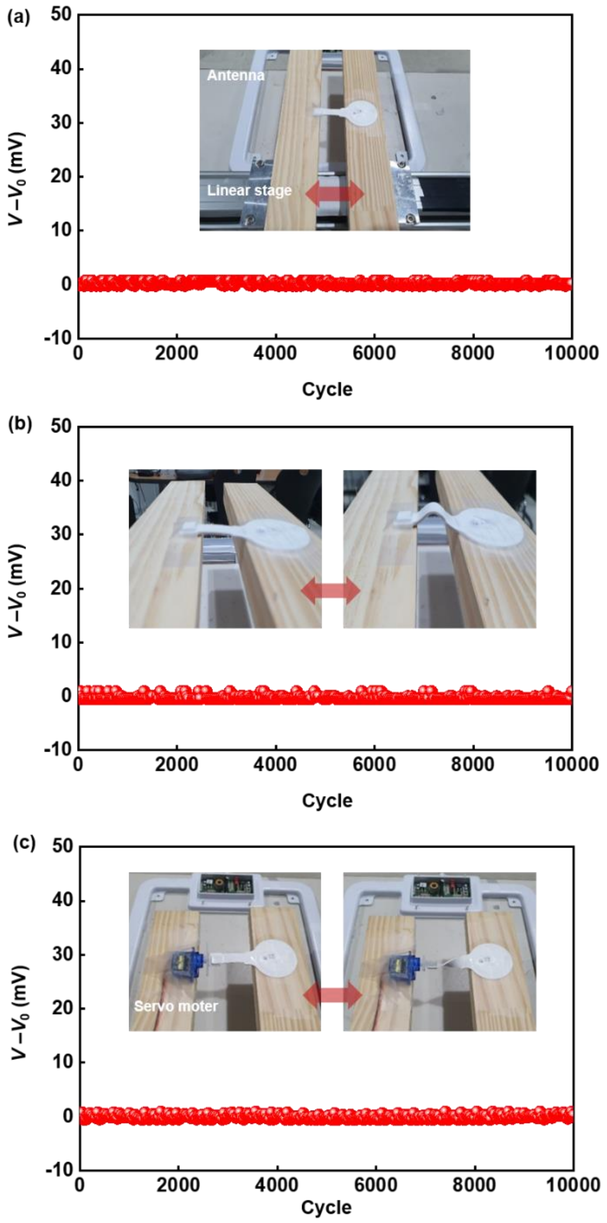

Supplementary Fig. 20. Electrical stability of the battery-free, wireless sensing platform. (a-c) Change of the ADC value from the NFC SoC under 10,000 repeated cycles of stretching (8%), of bending (7mm), of twisting (180°), respectively.

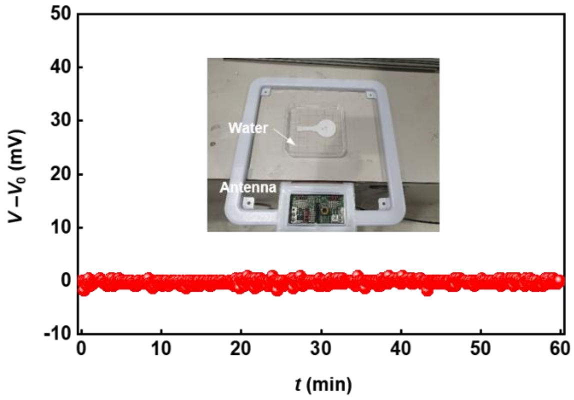

Supplementary Fig. 21. Change in the ADC value from the NFC SoC for a wireless device operating while completely submerged in water for 60 min.

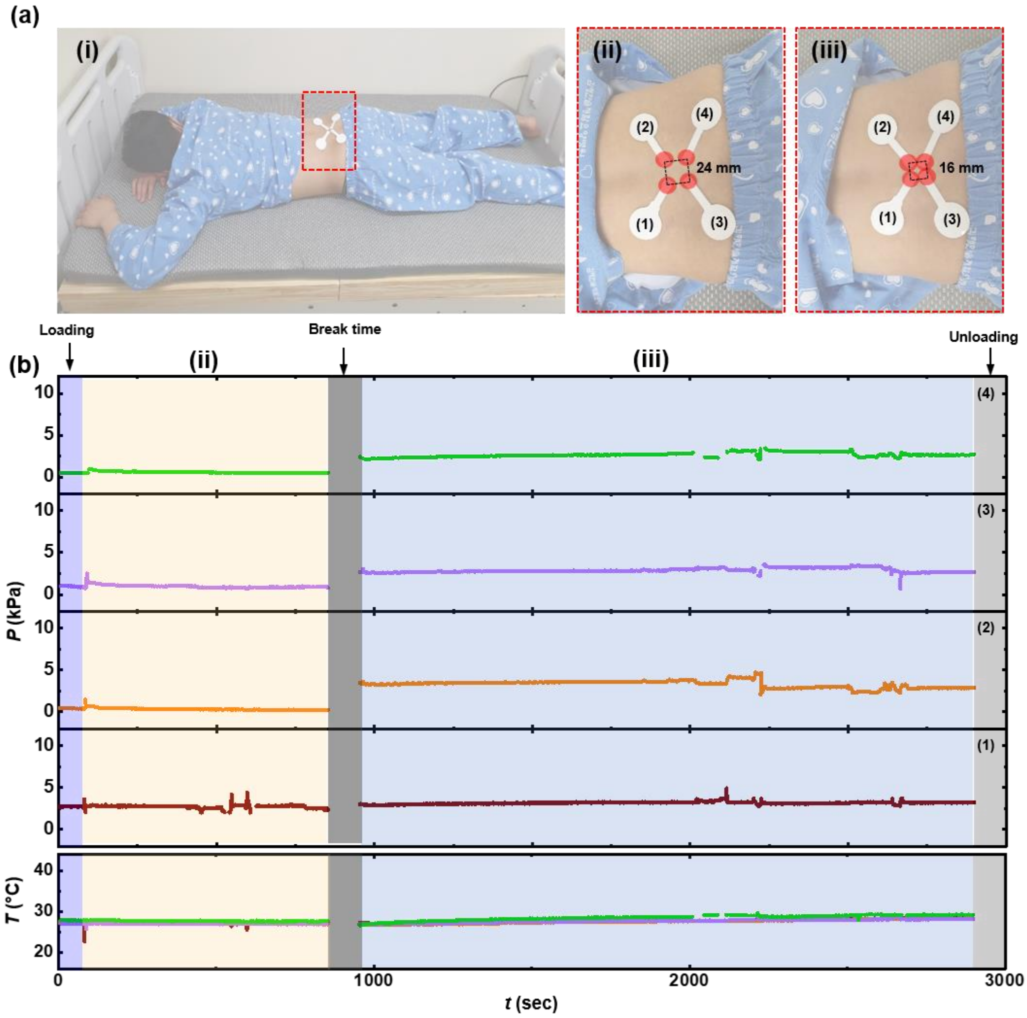

Supplementary Fig. 22. Continuous measurements of pressure and temperature from a healthy subject (30-year-old male, 72 kg, 180 cm) using 4 wireless sensing platforms placed in proximity to one another. (a) Photographs of mounting locations of sensors for the subject lying in prone position. (b) Pressure and temperature monitoring of the subject lying in supine position, respectively.

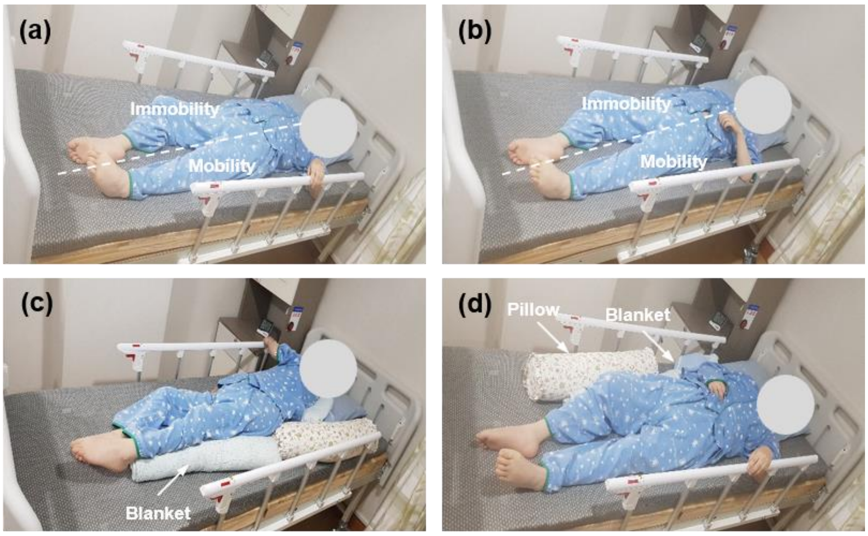

Supplementary Fig. 23. Photographic image of changes in posture of the subject with right hemiplegia. (a) An initial posture of the subject lying in bed. (b) Movement of a left arm by herself and fine movement at right side without the repositioning by the clinical staff. (c-d) changes in posture with the repositioning by the clinical staff using a pillow and a blanket.

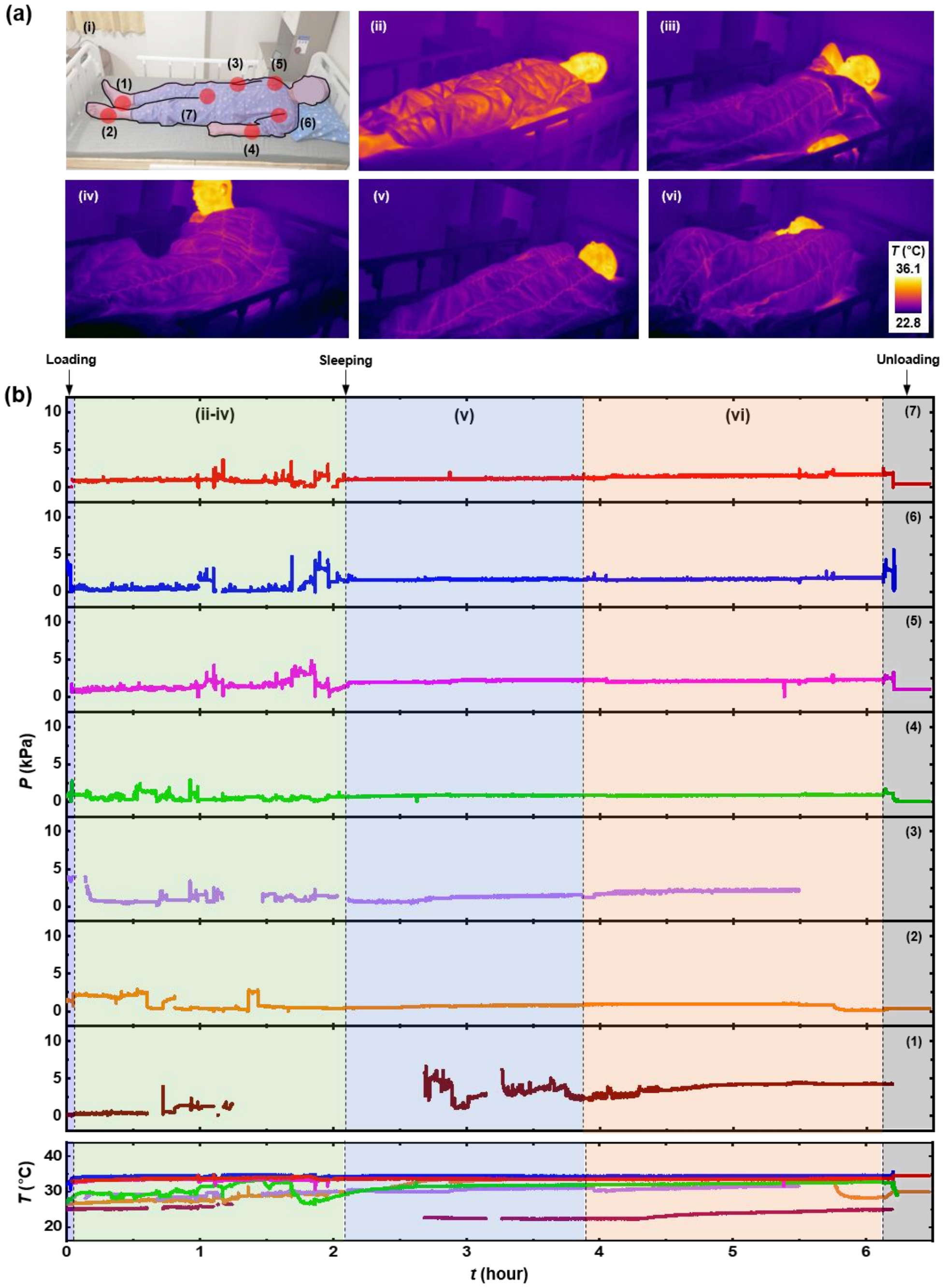

Supplementary Fig. 24. Continuous measurements of pressure and temperature from a subject with hemiplegia and stroke (61-year-old male, 57 kg, 170 cm) using the wireless sensing platform during sleep time (overnight). (a) Photograph of the subject with red discs to mark the mounting locations of the sensors and IR images of changes in posture of the subject lying on bed during sleep. (b) Results from continuous measurements of pressure and temperature from each of the sensors.

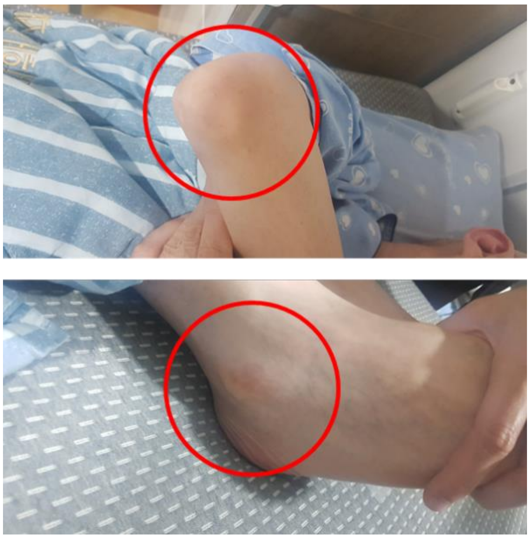

Supplementary Fig. 25. Photographic images of skin in the region of the right elbow and the heel after removing the wireless devices.

	No.	Protocol	Creat…	Comment	Process	Prt.No.	Time[…
A	1	02 00 12 FF BF 01 00 20 02 1F 02 DD FE 01 01 00 92 1C	no	Antenna 1	Autom...	-	2000
	2	02 00 14 FF B0 23 11 08 E0 07 A2 00 00 00 88 D5 09 02…	no	Sensor 1	Autom...	-	100
	3	02 00 14 FF B0 23 11 08 E0 07 A2 00 00 01 D3 CF 09 02…	no	Sensor 2	Autom...	-	100
	4	02 00 14 FF B0 23 11 08 E0 07 A2 00 00 00 88 DB 09 02…	no	Sensor 3	Autom...	-	100
	5	02 00 14 FF B0 23 11 08 E0 07 A2 00 00 01 DF 89 09 02…	no	Sensor 4	Autom...	-	100
	6	02 00 14 FF B0 23 11 08 E0 07 A2 00 00 01 DF 59 09 02…	no	Sensor 5	Autom...	-	100
	7	02 00 14 FF B0 23 11 08 E0 07 A2 00 00 01 F8 45 09 02…	no	Sensor 6	Autom...	-	100
	8	02 00 14 FF B0 23 11 08 E0 07 A2 00 00 01 D3 9B 09 02…	no	Sensor 7	Autom...	-	100
	9	02 00 14 FF B0 23 11 08 E0 07 A2 00 00 00 88 D9 09 02…	no	Sensor 8	Autom...	-	100
	10	02 00 14 FF B0 23 11 08 E0 07 A2 00 00 01 D3 C6 09 02…	no	Sensor 9	Autom...	-	100
	11	02 00 14 FF B0 23 11 08 E0 07 A2 00 00 01 DF 86 09 02…	no	Sensor 10	Autom...	-	100
B	12	02 00 12 FF BF 01 00 20 02 1F 02 DD FE 01 02 00 FA 36	no	Antenna 2	Autom...	-	2000
	13	02 00 14 FF B0 23 11 08 E0 07 A2 00 00 00 88 D5 09 02…	no	Sensor 1	Autom...	-	100
	14	02 00 14 FF B0 23 11 08 E0 07 A2 00 00 01 D3 CF 09 02…	no	Sensor 2	Autom...	-	100
	15	02 00 14 FF B0 23 11 08 E0 07 A2 00 00 00 88 DB 09 02…	no	Sensor 3	Autom...	-	100
	16	02 00 14 FF B0 23 11 08 E0 07 A2 00 00 01 DF 89 09 02…	no	Sensor 4	Autom...	-	100
	17	02 00 14 FF B0 23 11 08 E0 07 A2 00 00 01 DF 59 09 02…	no	Sensor 5	Autom...	-	100
	18	02 00 14 FF B0 23 11 08 E0 07 A2 00 00 01 F8 45 09 02…	no	Sensor 6	Autom...	-	100
	19	02 00 14 FF B0 23 11 08 E0 07 A2 00 00 01 D3 9B 09 02…	no	Sensor 7	Autom...	-	100
	20	02 00 14 FF B0 23 11 08 E0 07 A2 00 00 00 88 D9 09 02…	no	Sensor 8	Autom...	-	100
	21	02 00 14 FF B0 23 11 08 E0 07 A2 00 00 01 D3 C6 09 02…	no	Sensor 9	Autom...	-	100
	22	02 00 14 FF B0 23 11 08 E0 07 A2 00 00 01 DF 86 09 02…	no	Sensor 10	Autom...	1	100

Supplementary Fig. 26. Programming of ISOStart2018 software in protocol mode for operation of multiplexed antenna and multiple sensors. Commands of A and B mean sequentially turning on antenna 1 and antenna 2, respectively.

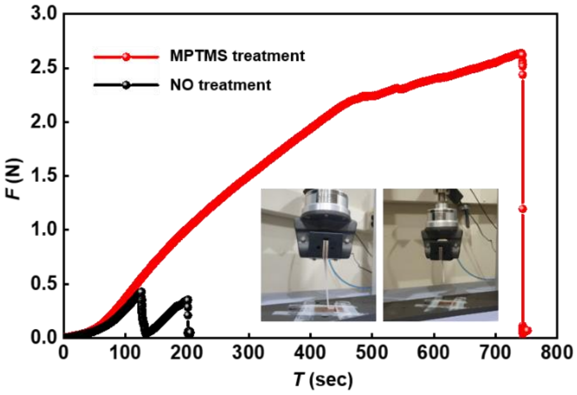

Supplementary Fig. 27. Comparison of adhesive strength between the MPTMS-treated silicone (Dragon Skin) and the PI film for epoxy bonding. The insets show photographic images of samples of dragon skin peeling from a PI film under tensile loading.

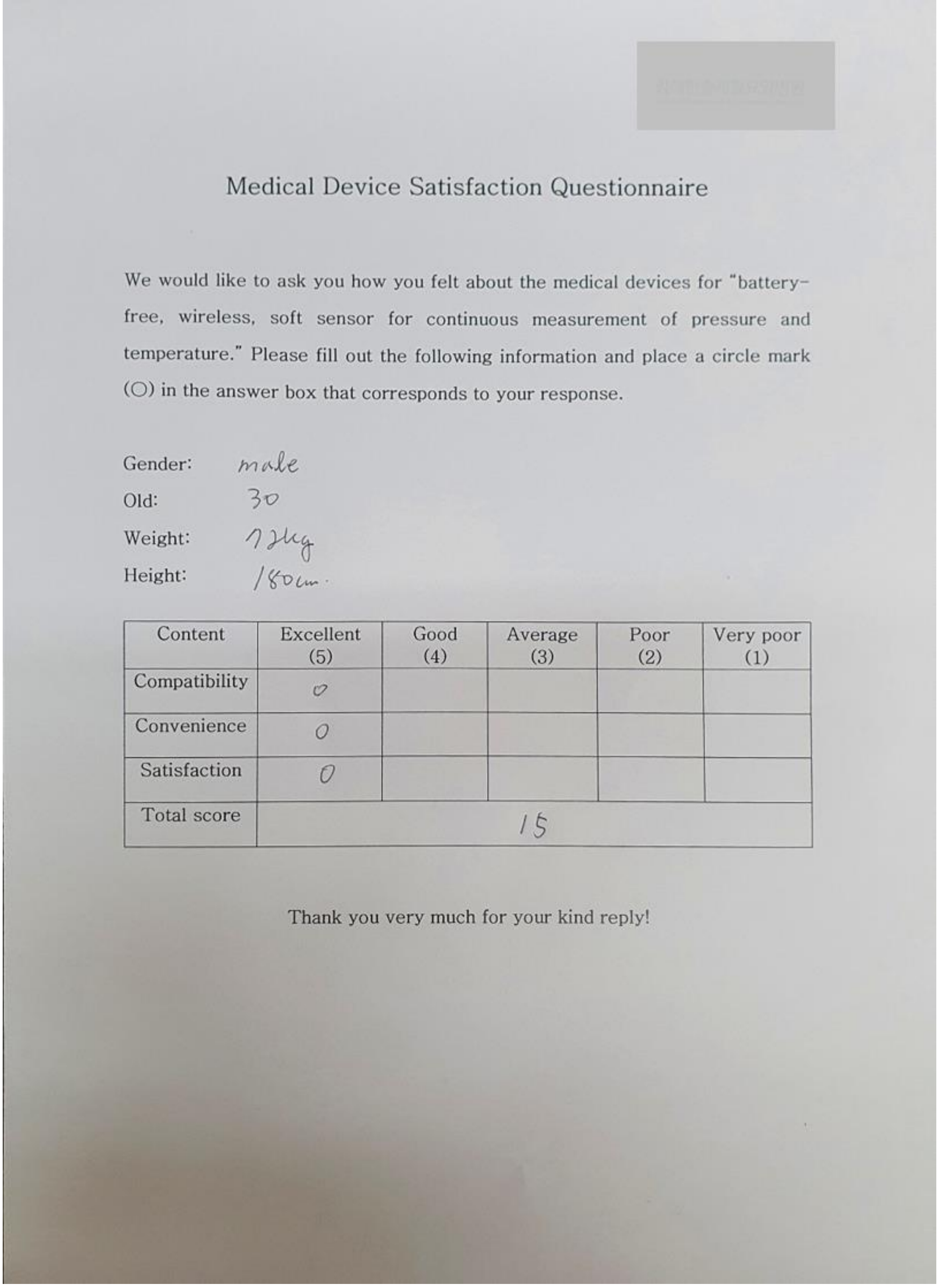

[illegible]

Medical Device Satisfaction Questionnaire

We would like to ask you how you felt about the medical devices for "battery-free, wireless, soft sensor for continuous measurement of pressure and temperature." Please fill out the following information and place a circle mark (O) in the answer box that corresponds to your response.

Gender: male
Old: 30
Weight: 72kg
Height: 180cm.

Content	Excellent (5)	Good (4)	Average (3)	Poor (2)	Very poor (1)
Compatibility	O				
Convenience	O				
Satisfaction	O				
Total score	15				

Thank you very much for your kind reply!

Supplementary Fig. 28. Feedback from the healthy subject (30-year-old male, 72 kg, 180 cm) in Fig. 5.

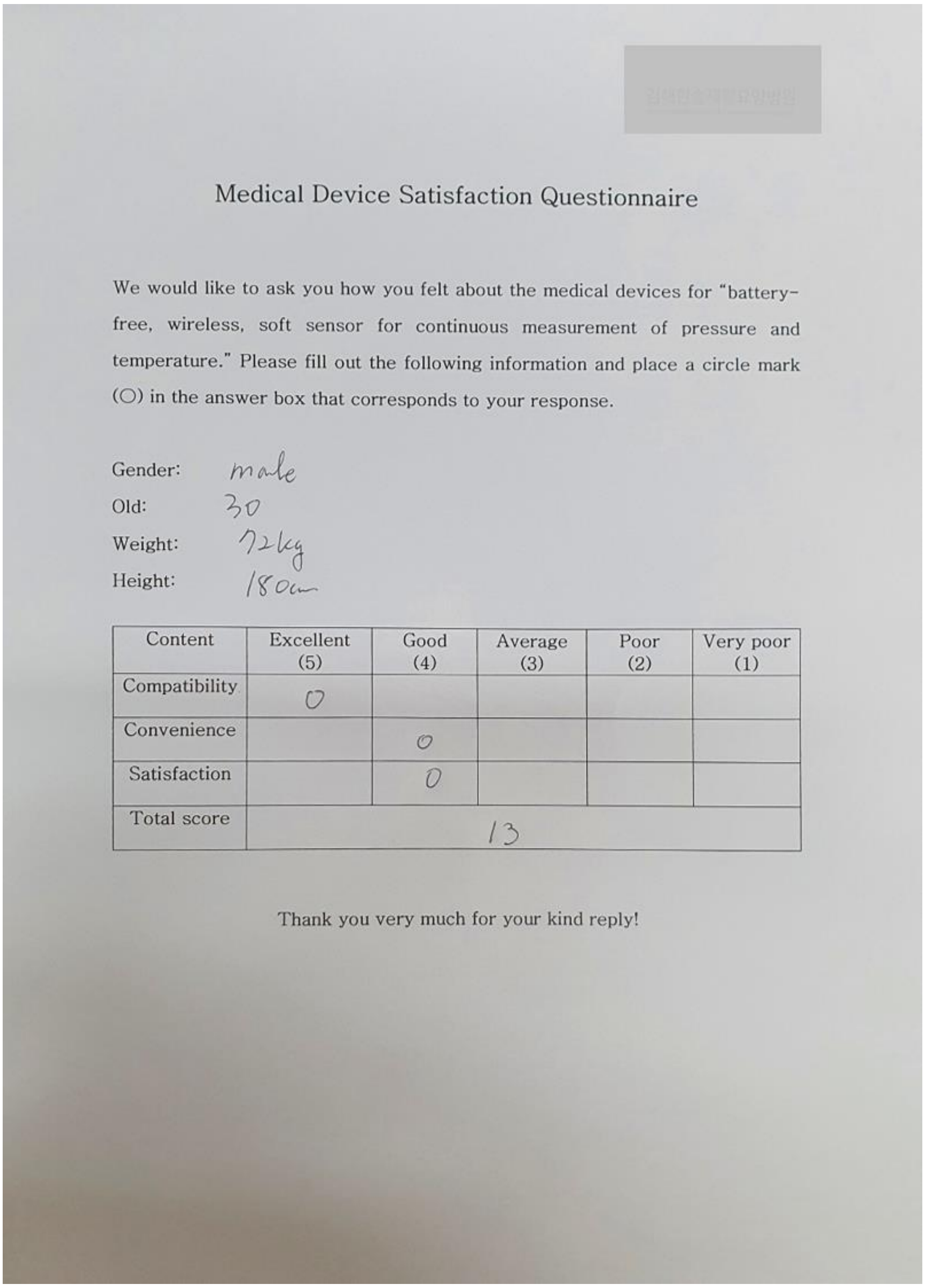

[illegible]

Medical Device Satisfaction Questionnaire

We would like to ask you how you felt about the medical devices for "battery-free, wireless, soft sensor for continuous measurement of pressure and temperature." Please fill out the following information and place a circle mark (O) in the answer box that corresponds to your response.

Gender: male
Old: 30
Weight: 72kg
Height: 180cm

Content	Excellent (5)	Good (4)	Average (3)	Poor (2)	Very poor (1)
Compatibility	O				
Convenience		O			
Satisfaction		O			
Total score	13				

Thank you very much for your kind reply!

Supplementary Fig. 29. Feedback from the healthy subject (30-year-old male, 72 kg, 180 cm) in Fig. 6.

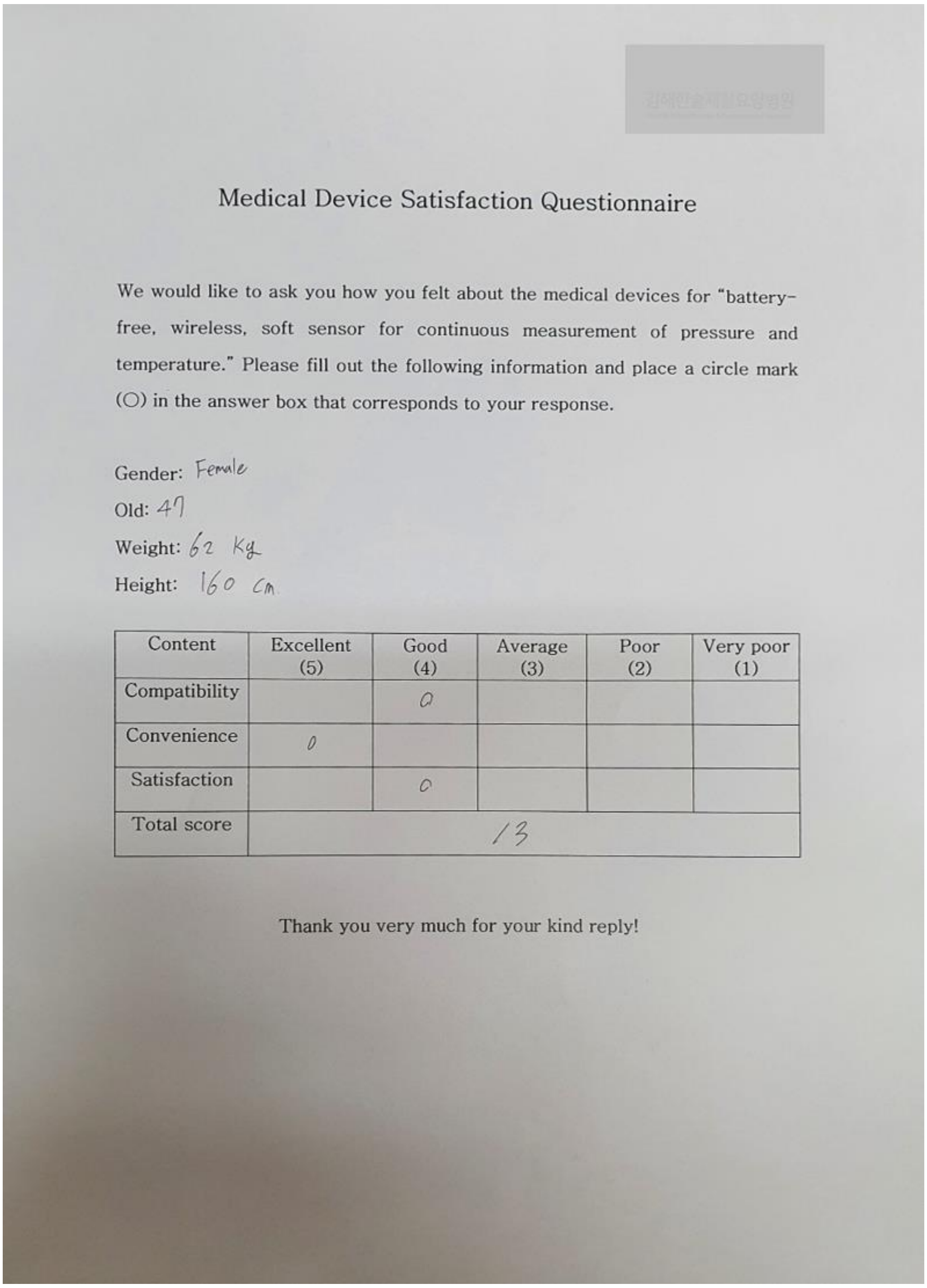

Medical Device Satisfaction Questionnaire

We would like to ask you how you felt about the medical devices for "battery-free, wireless, soft sensor for continuous measurement of pressure and temperature." Please fill out the following information and place a circle mark (O) in the answer box that corresponds to your response.

Gender: Female
Old: 47
Weight: 62 Kg
Height: 160 Cm

Content	Excellent (5)	Good (4)	Average (3)	Poor (2)	Very poor (1)
Compatibility		O			
Convenience	O				
Satisfaction		O			
Total score	13				

Thank you very much for your kind reply!

Supplementary Fig. 30. Feedback from the patient with right hemiplegia (47-year-old female, 62 kg, 160 cm) in Fig. 7.

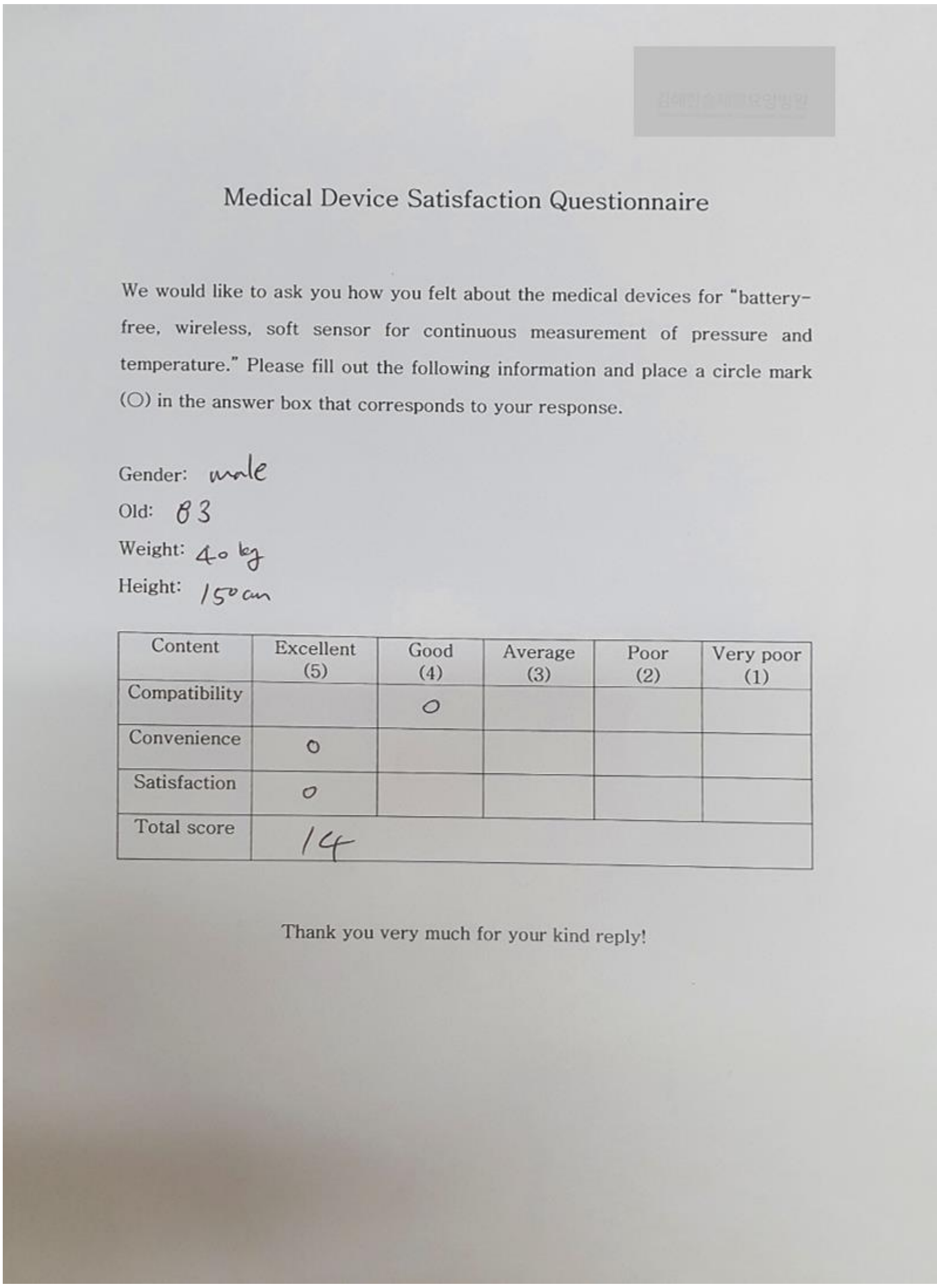

Medical Device Satisfaction Questionnaire

We would like to ask you how you felt about the medical devices for "battery-free, wireless, soft sensor for continuous measurement of pressure and temperature." Please fill out the following information and place a circle mark (○) in the answer box that corresponds to your response.

Gender: male
Old: 83
Weight: 40 kg
Height: 150 cm

Content	Excellent (5)	Good (4)	Average (3)	Poor (2)	Very poor (1)
Compatibility		o			
Convenience	o				
Satisfaction	o				
Total score	14				

Thank you very much for your kind reply!

Supplementary Fig. 31. Feedback from the patient with general paralysis (83-year-old male, 40 kg, 150 cm) in Fig. 8.

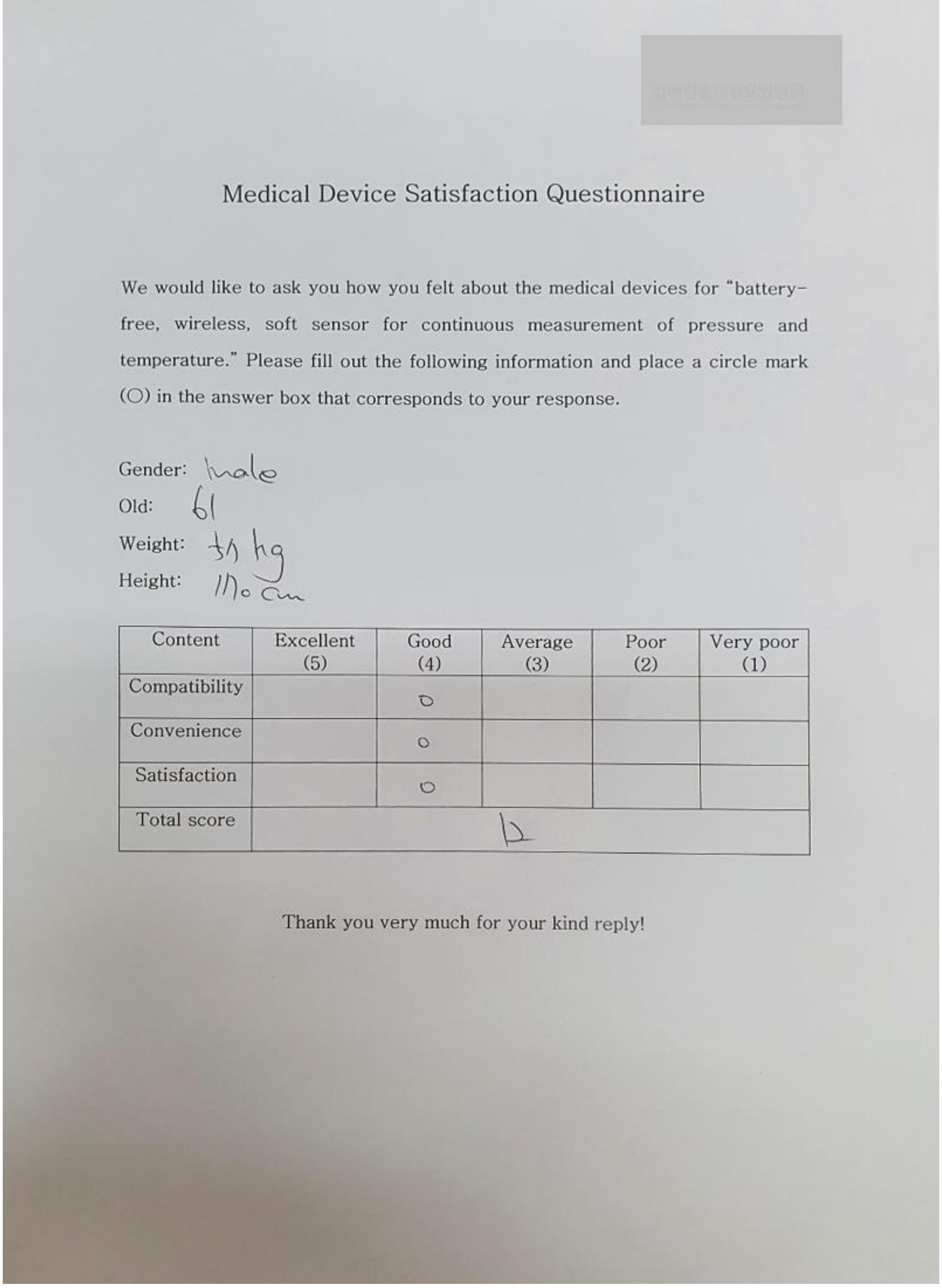

Medical Device Satisfaction Questionnaire

We would like to ask you how you felt about the medical devices for "battery-free, wireless, soft sensor for continuous measurement of pressure and temperature." Please fill out the following information and place a circle mark (○) in the answer box that corresponds to your response.

Gender: male
Old: 61
Weight: 57 kg
Height: 170 cm

Content	Excellent (5)	Good (4)	Average (3)	Poor (2)	Very poor (1)
Compatibility		o			
Convenience		o			
Satisfaction		o			
Total score		12			

Thank you very much for your kind reply!

Supplementary Fig. 32. Feedback from the patient with hemiplegia and stroke (61-year-old male, 57 kg, 170 cm) in Supplementary Fig. 24.

Supplementary Table 1. Comparison for battery-free, wireless sensors.

	Content	This study	Previous study
Pressure sensor	Sensing mechanism	Bending-induced tensile strain (membrane deflection)	Stretching-induced tensile strain (Poisson effect of PDMS)
	Resistive material	Au	P-doped, Si membrane
	Gauge factor	~2	~50
	Sensitivity	< 0.0006 kPa^{-1}	<0.002 kPa^{-1}
	Bending	Insensitive	Sensitive
	Shear	Insensitive	No experimental data
	Robustness	Good (Stability under cyclic loading)	Not good (No data under cyclic loading)
	Reproducibility	Good	Not good
	Temp. Compensation	Yes (using NTC)	No experimental data
Wireless platform	NFC chip (ADC)	RF430FRL152H, Texas Instruments (3 counts)	SL13A, amg AG; (1 count)
	Maximum working distance @diameter of coil	46 cm @ 34.5 mm	32cm @16 mm
	Location of sensor	Out of receiver antenna (with serpentine trace)	In receiver antenna
	Connection type	Wheatstone bridge	Voltage divider
	Software	ISOStart 2018	-
Clinical trial	Patient at risk for pressure injuries	Yes (Two hemiplegic patients, one tetraplegic patient)	No
	Simultaneous monitoring of pressure and temperature	Yes	No
	Continuous monitoring for change in posture	Yes	No

Supplementary References

1. Han, S. *et al.* Battery-free, wireless sensors for full-body pressure and temperature mapping. *Sci. Transl. Med.* **10**, eaan4950 (2018).
2. Swain, I. D. & Bader, D. L. The measurement of interface pressure and its role in soft tissue breakdown. *J. Tissue Viability* **12**, 132–146 (2002).
3. Liao, F., Burns, S. & Jan, Y. K. Skin blood flow dynamics and its role in pressure ulcers. *J. Tissue Viability* **22**, 25–36 (2013).
4. Kobara, K. *et al.* Mechanism of fluctuation in shear force applied to buttocks during reclining of back support on wheelchair. *Disabil. Rehabil. Assist. Technol.* **8**, 220–224 (2013).
5. Smith, S., Snyder, A., McMahon, L. F., Petersen, L. & Meddings, J. Success in hospital-acquired pressure ulcer prevention: A tale in two data sets. *Health Aff.* **37**, 1787–1796 (2018).
6. Fard, F. D., Moghimi, S. & Lotfi, R. Evaluating Pressure Ulcer Development in Wheelchair-Bound Population Using Sitting Posture Identification. *Engineering* **5**, 132–136 (2013).
7. Sprigle, S., Sonenblum, S. E. & Feng, C. Pressure redistributing in-seat movement activities by persons with spinal cord injury over multiple epochs. *PLoS One* **14**, e0210978 (2019).
8. Shin, G. *et al.* Flexible Near-Field Wireless Optoelectronics as Subdermal Implants for Broad Applications in Optogenetics. *Neuron* **93**, 509–521 (2017).
9. Frantz, R. A. & Xakellis, G. C. Characteristics of skin blood flow over the trochanter under constant, prolonged pressure. *Am. J. Phys. Med. Rehabil.* **68**, 272–276 (1989).
10. Grap, M. J. *et al.* Tissue interface pressure and skin integrity in critically ill, mechanically ventilated patients. *Intensive Crit. Care Nurs.* **38**, 1–9 (2017).
11. Kokate, J. Y. *et al.* Temperature-modulated pressure ulcers: A porcine model. *Arch. Phys. Med. Rehabil.* **76**, 666–673 (1995).
12. Schwartz, D., Magen, Y. K., Levy, A. & Gefen, A. Effects of humidity on skin friction against medical textiles as related to prevention of pressure injuries. *Int. Wound J.* **15**, 866–874 (2018).
